# A Method to Quantify FRET Stoichiometry with Phasor Plot Analysis and Acceptor Lifetime Ingrowth

**DOI:** 10.1016/j.bpj.2015.01.012

**Published:** 2015-03-10

**Authors:** WeiYue Chen, Edward Avezov, Simon C. Schlachter, Fabrice Gielen, Romain F. Laine, Heather P. Harding, Florian Hollfelder, David Ron, Clemens F. Kaminski

**Affiliations:** 1 Department of Chemical Engineering and Biotechnology, University of Cambridge, Cambridge, United Kingdom; 2 The Wellcome Trust Medical Research Council Institute of Metabolic Science and National Institute for Health Research, Cambridge Biomedical Research Centre, Cambridge Institute for Medical Research, University of Cambridge, Cambridge, United Kingdom; 3 Department of Biochemistry, University of Cambridge, Cambridge, United Kingdom

## Abstract

FRET is widely used for the study of protein-protein interactions in biological samples. However, it is difficult to quantify both the FRET efficiency (*E*) and the affinity (*K*_*d*_) of the molecular interaction from intermolecular FRET signals in samples of unknown stoichiometry. Here, we present a method for the simultaneous quantification of the complete set of interaction parameters, including fractions of bound donors and acceptors, local protein concentrations, and dissociation constants, in each image pixel. The method makes use of fluorescence lifetime information from both donor and acceptor molecules and takes advantage of the linear properties of the phasor plot approach. We demonstrate the capability of our method in vitro in a microfluidic device and also in cells, via the determination of the binding affinity between tagged versions of glutathione and glutathione S-transferase, and via the determination of competitor concentration. The potential of the method is explored with simulations.

## Main Text

Förster resonance energy transfer (FRET) is widely applied in the study of molecular interactions and conformational changes in biological systems (1). Both the FRET efficiency, *E*, and the fraction of molecules participating in the interaction, *f*, are important parameters in biochemical research. A number of intensity-based FRET methods have been developed to quantify *E* and *f* (2,3). Those can be performed with basic fluorescence equipment, which is advantageous; but they also require extensive calibration protocols, which may lead to large cumulative errors.

Fluorescence lifetime imaging microscopy (FLIM) provides a more robust means of quantifying FRET interactions because the fluorescence lifetime is an inherently ratiometric measurement (4–6). In existing FLIM methods, the fluorescence decay can be analyzed either by decay-curve fitting (7) or by using a geometric global analysis approach, called the AB- (8,9) or phasor-plot method (10–14). Both FRET efficiency and molecular fractions of active donors (i.e., donors participating in the FRET process), *f*_*D*_^FRET^, can be recovered. The value *f*_*D*_^FRET^ depends on several factors, such as local concentrations of donor and acceptor and the binding affinity between them. All of these are of interest, but they cannot be quantified without knowledge of the bound acceptor fraction (fraction of acceptors that are in complex with their binding partners), which is not traditionally available when only donor lifetimes are measured. Spectrally resolved FLIM has been applied for FRET measurements to improve both the separation of multiple lifetime components and the accuracy of recovered FRET efficiencies (6,15–17), but they have not been extended to the recovery of the acceptor stoichiometry. The lifetime ingrowth of acceptors has been exploited for the analysis of FRET stoichiometry (18,19); however, these methods are impractical when fluorescence bleedthrough from donor fluorophores contaminates the FRET signal, a problem for most FRET pairs, because then the bound acceptor fraction becomes difficult to retrieve.

Here, we present a method, which combines the advantages of FLIM and phasor plot techniques, taking into full account the presence of cross-excitation (direct excitation of the acceptor upon donor excitation) and donor fluorescence bleedthrough in the acceptor emission channel. FRET efficiency and molecular fractions of both the bound donor and acceptor molecules are recovered, as well as the dissociation constant *K*_*d*_. Measurements in a maximum of only three spectral channels are required by our method, which we refer to as multichannel FLIM-FRET (MC-FLIM-FRET). The validity and potential of the method are explored with simulations, and demonstrated experimentally with time-correlated single photon counting measurements in microfluidic devices and in cells. We quantified the binding affinity between glutathione (GSH) fused to the fluorescein derivative Oregon green (OG-GSH, donor) and glutathione S-transferase (GST) fused to the fluorescent protein mCherry (mCherry-GST, acceptor) for various protein stoichiometries (for details on constructs, see Section S2 in the Supporting Material).

**Figure 1:**
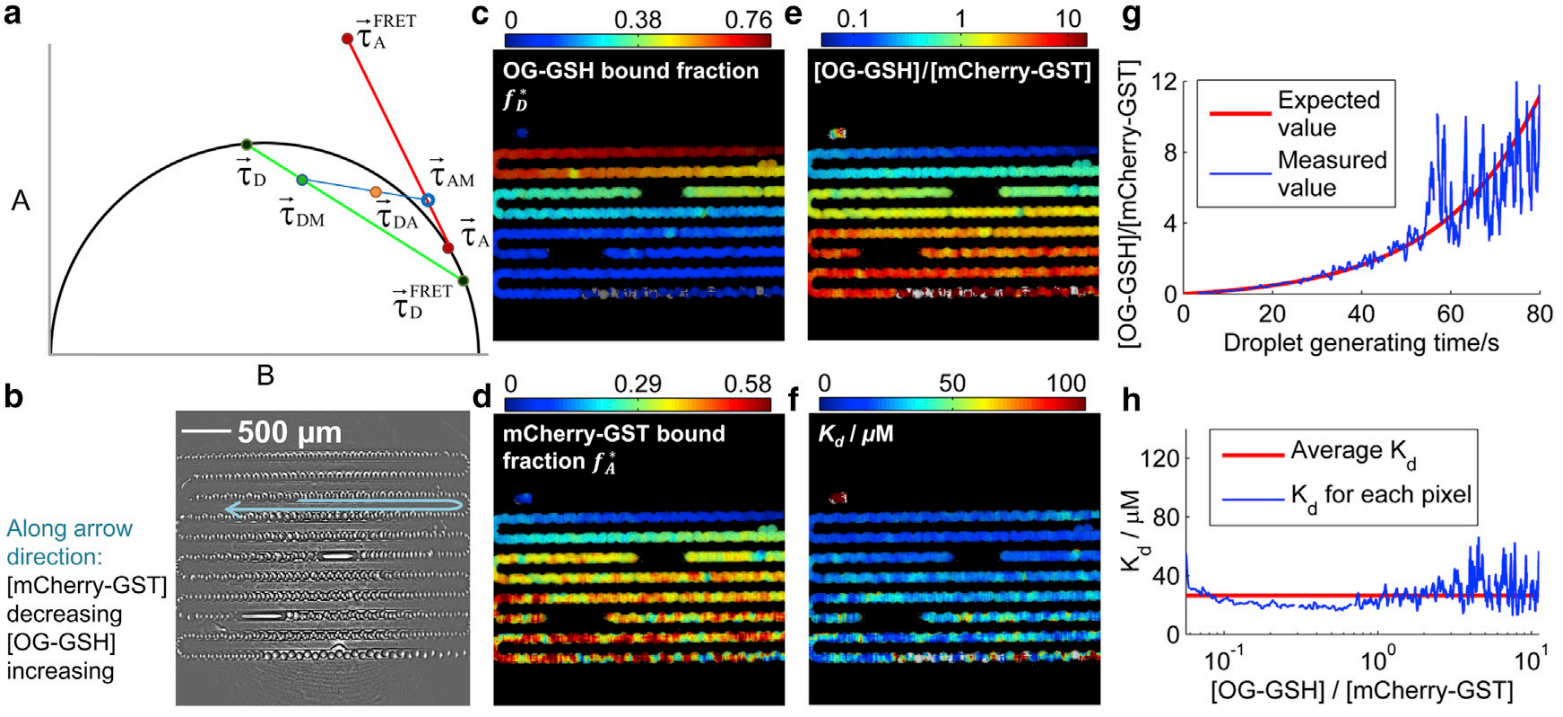
Principle of MC-FLIM-FRET, and validation. (*a*) Explanation of phasor plot construction for MC-FLIM-FRET. (*b*) Transmitted light image of a microfluidic device containing a sequence of microdroplets with continuously varying stoichiometry. (*c*) Recovered fraction of bound donor. (*d*) Recovered fraction of bound acceptor. (*e*) Recovered concentration ratio between donor and acceptor (log scale). (*f*) Recovered dissociation constant *K*_*d*_. (*g*) Recovered concentration ratio between donor and acceptor (*blue line*), and expected value calculated from known mixing conditions during droplet generation. (*h*) The value *K*_*d*_ is verified to be independent of [D]/[A]. The average photon count in each binned pixel is ∼14,000 for panels *c–f*, and ∼90,000 for *g* and *h* (see Section S3 in the Supporting Material for details).

Fig. 1 *a* shows the principle of MC-FLIM-FRET. The method requires the measurement of fluorescence decays in both the donor channel (donor excitation/donor emission) and the FRET channel (donor excitation/acceptor emission). Here the case for fluorophores exhibiting monoexponential decays is discussed. Multiexponential decays are discussed in Section S1 in the Supporting Material. Fluorescence measured in the donor channel only contains a mixture of signal from donors participating in FRET (active donors) and those that do not (passive donors) (3,6), hence the corresponding mixed phasor $\left({\vec{\tau }}_{DM}\right)$ lies along the line joining the phasors of active $\left({\vec{\tau }}_{D}^{\text{FRET}}\right)$ and passive donors $\left({\vec{\tau }}_{D}\right)$. From the positions of ${\vec{\tau }}_{DM}$, both the active donor fractions, *f*_*D*_^FRET^, and FRET efficiency, *E*, can be recovered as previously demonstrated ((10,11), and see Section S1 in the Supporting Material). The bound donor fraction *f*_*D*_^∗^ is the same as *f*_*D*_^FRET^ (see Section S1 in the Supporting Material for detail).

On the other hand, the phasor for the FRET channel, ${\vec{\tau }}_{DA}$, is a linear combination of active and passive acceptor phasors (${\vec{\tau }}_{A}^{\mathrm{FRET}}$ and ${\vec{\tau }}_{A}$, respectively) and ${\vec{\tau }}_{DM}$ (combination of ${\vec{\tau }}_{D}^{\text{FRET}}$ and ${\vec{\tau }}_{D}$) resulting from donor bleedthrough. The value ${\vec{\tau }}_{A}$ is easily obtained from a FLIM measurement in the acceptor channel (acceptor excitation/acceptor emission) using a sufficiently long excitation wavelength, or, alternatively, via measurement in a control sample containing acceptors only. The value ${\vec{\tau }}_{A}^{\text{FRET}}$ is calculated from ${\vec{\tau }}_{A}$ and ${\vec{\tau }}_{D}^{\text{FRET}}$ by considering the acceptor lifetime ingrowth using the methods detailed in Section S1 in the Supporting Material. The phasor ${\vec{\tau }}_{AM}$ (containing only the contribution from acceptors) can then be obtained by the intersection of the line ${\vec{\tau }}_{DM}$ − ${\vec{\tau }}_{DA}$ (*blue line* in Fig. 1) with the acceptor phasor trajectory (*red line*), from which the fraction of FRET active acceptors *f*_*A*_^FRET^ can finally be determined. Note that, due to cross-excitation, not all of the acceptor molecules bound to donor molecules are FRET-active. The fraction of bound acceptors *f*_*A*_^∗^ can be recovered with *f*_*A*_^FRET^ using the methods described in Section S1 in the Supporting Material. Hence, both FRET efficiency and stoichiometry are resolved with our method. If either donor concentration ([D]) or acceptor concentration ([A]) is known a priori, then *K*_*d*_ can also be recovered. If this is not the case, then [A] can be recovered from an intensity measurement in the acceptor channel. (See Section S1 in the Supporting Material for further explanations.)

To explore the dynamic range of MC-FLIM-FRET, we performed simulations using spectral parameters mimicking the OG/mCherry pair and eGFP/mCherry pair (see Section S4 in the Supporting Material for details). The simulations were performed in the presence of realistic levels of noise, and verify that donor- and acceptor-bound fractions as well as *K*_*d*_ can be recovered with good accuracy from data with signal levels typically available in real experiments.

Next, we validated the method experimentally by imaging a microfluidic device filled with ∼500 microdroplets of volume 3 nL, each containing a unique stoichiometry of OG-GSH and mCherry-GST with [D]/[A] ranging from 0.06 to 11.19 (see Section S2 in the Supporting Material for details). Fig. 1 *b* shows a transmitted light image of the microfluidic device. Fig. S13 a in the Supporting Material shows the corresponding phasor plot for the data from which a FRET efficiency of *E* = (59.5 ± 0.5)% (errors quoted as SE, 68% confidence interval) is recovered. Fig. 1, *c* and *d*, presents the recovered bound fractions *f*_*D*_^∗^ and *f*_*A*_^∗^, respectively, from which we obtain the [D]/[A] across the image (Fig. 1 *e*, and see Section S1 in the Supporting Material for details). Fig. 1 *f* shows the recovered dissociation constant *K*_*d*_ across the image. Because each spatial position inside the microfluidic device correlates with the time point when the mixture was generated, we can plot the temporal evolution of the concentration ratios measured (*blue curve*, Fig. 1 *g*) and compare this with the known values (*red curve*; see Section S2 in the Supporting Material for details). The data are in good agreement. Fig. 1 *h* shows the recovered *K*_*d*_ value for different donor and acceptor concentration ratios and as expected, the recovered *K*_*d*_ value is approximately constant throughout. We obtain a mean value of *K*_*d*_ = 26.5 ± 0.2 *μ*M. Fig. 1, *g* and *h*, shows how the sensitivity of the method decreases as [D]/[A] gets large, and signal/noise correspondingly small. In Section S4 in the Supporting Material we compare the experimental noise performance and measurement sensitivity with simulations, and both are in good agreement.

**Figure 2:**
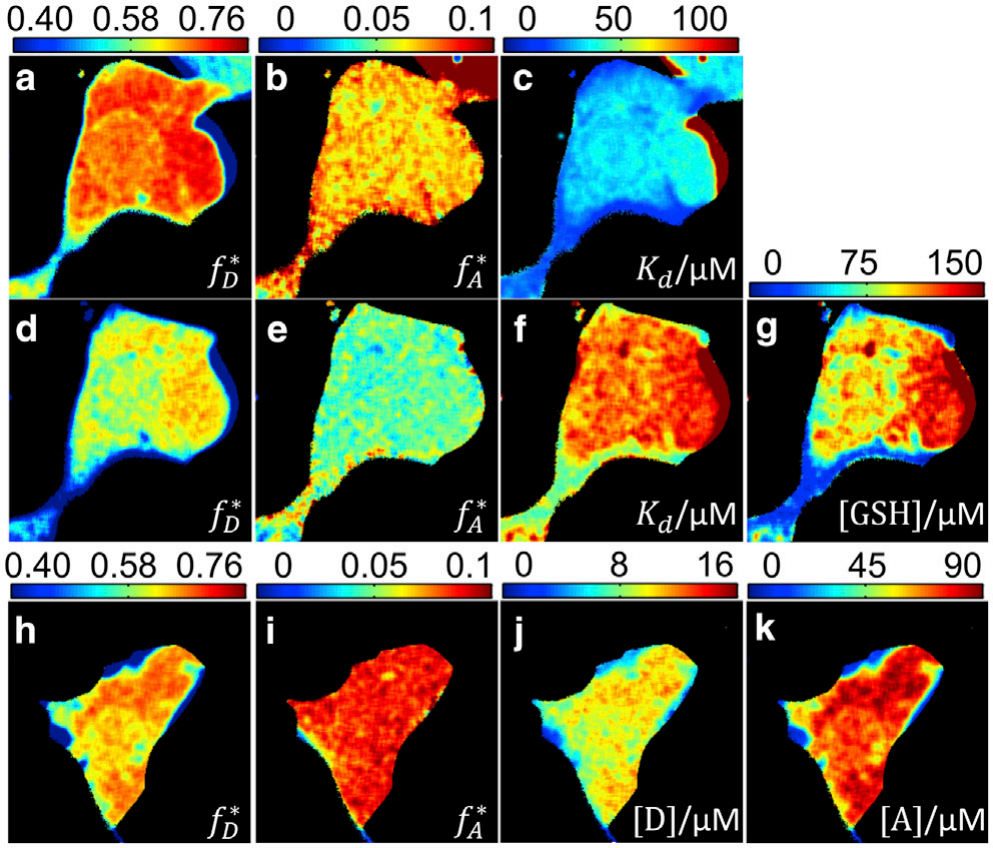
Validation of MC-FLIM-FRET in cells. (*a–c*) Bound fractions and dissociation constants. (*d–g*) Recovered parameters upon adding competitor GSH. (*h–k*) Absolute concentration determination in cells with known *K*_*d*_ = 37.2 ± 0.2 *μ*M. The average photon count in each binned pixel is ∼13,000 (see Section S3 in the Supporting Material for details).

We also tested the performance of the method for measurements in cells, with autofluorescence taken into account (20). HEK293T cells expressing mCherry-GST were prepared and permeabilized with saponin, a mild detergent (21). OG-GSH was then added to the medium and its diffusion ensued into the cells. The cell-endogenous GSH, which is a competitor for the OG-GSH and mCherry-GST interaction, was depleted after membrane permeabilization (Section S2 in the Supporting Material). Fig. 2, *a* and *b*, shows the bound fractions of donors and acceptors, respectively, for a representative cell. The recovered FRET efficiency is (58.7 ± 0.6)%. Using a further measurement in the acceptor channel, we recovered the acceptor concentration [A] (see Section S3 in the Supporting Material), and hence, *K*_*d*_, as shown in Fig. 2 *c*. We obtain an average value of *K*_*d*_ = 37.2 ± 0.2 *μ*M. Although similar to the microdroplet result, the difference is likely to reflect the residual presence of endogenous GSH and the different solution conditions prevailing in the cell. Next, we added 200 *μ*M GSH to the medium to introduce the effect of a competitor. Fig. 2, *d* and *e*, shows the recovered *f*_*D*_^∗^ and *f*_*A*_^∗^ in this case; both are lower than in absence of competitor, as expected. The calculated apparent *K*_*d*_, Fig. 2 *f*, is now clearly larger than in the GSH-depleted sample shown in Fig. 2 *c*. Assuming that the real *K*_*d*_ value is unchanged, we can now recover the concentration of the competitor, GSH (Fig. 2 *g*). We thus obtain a GSH concentration of 93.3 ± 0.3 *μ*M. This reduced concentration is likely reflective of the fact that GSH undergoes oxidation during sample preparation (Section S2 in the Supporting Material). Finally, even in the case where neither donor nor acceptor concentrations are available, it is possible to recover variations in *K*_*d*_ and competitor concentrations across a sample (see Section S1 in the Supporting Material for details).

On the other hand, for a known *K*_*d*_ value in a bimolecular complex, and in the absence of competitor reactions, both absolute donor and acceptor concentrations can be recovered (Section S1 in the Supporting Material). In Fig. 2, *h* and *i*, the bound fractions *f*_*D*_^∗^ and *f*_*A*_^∗^ are presented for another cell. Assuming a *K*_*d*_ value as was measured in Fig. 2 *c*, [D] and [A] can be recovered in the cell (Fig. 2, *j* and *k*). The average [A] recovered in this way is (66.9 ± 0.2) *μ*M, which compares well with an acceptor-intensity-based measurement of (50.8 ± 0.1) *μ*M, giving confidence to both the robustness of the method and the extracted value for *K*_*d*_.

In summary, we have developed a robust method for FRET quantification using FLIM measurements in both the donor and acceptor emission channels, in combination with a powerful phasor plot approach. It permits us to compensate for donor bleedthrough and acceptor cross-excitation, recovering both FRET efficiency and molecular fractions of bound donor and acceptor complexes, unachievable with common FLIM-FRET techniques. The method was validated using simulation, microfluidic experiments, and cell experiments. Our method is useful for measurements of dissociation constants, donor and acceptor concentrations, and the presence and concentration of competitors to binding reactions.

## Author Contributions

W.Y.C. designed and performed the research, analyzed the data, and wrote the manuscript. E.A. contributed tools and performed the research. S.C.S. contributed analytic tools and performed the research. F.G. contributed tools and performed the research. R.F.L. analyzed the data. H.P.H. performed the research. F.H. contributed tools. D.R. designed the research and contributed tools. C.F.K. designed the research, supervised the project, and wrote the manuscript.

## Supporting Material

Document S1. Supporting Materials and Methods, 13 figures, and one table

Document S2. Article plus Supporting Material

## Supporting Citations

References (22–32) appear in the Supporting Material.
