# Supplementary material for: A Method to Quantify FRET Stoichiometry with Phasor Plot Analysis and Acceptor Lifetime Ingrowth
Source: Biophys J. 2015 Mar 10;108(5):999–1002. doi: 10.1016/j.bpj.2015.01.012 (PMC4375440; doi:10.1016/j.bpj.2015.01.012)
Supplement: Document S2. Article plus Supporting Material [file mmc2.pdf]

Biophysical Letter

A Method to Quantify FRET Stoichiometry with Phasor Plot Analysis and Acceptor Lifetime Ingrowth

WeiYue Chen,[1] Edward Avezov,[2] Simon C. Schlachter,[1] Fabrice Gielen,[3] Romain F. Laine,[1] Heather P. Harding,[2] Florian Hollfelder,[3] David Ron,[2] and Clemens F. Kaminski[1,*]
[1]Department of Chemical Engineering and Biotechnology, [2]The Wellcome Trust Medical Research Council Institute of Metabolic Science and National Institute for Health Research, Cambridge Biomedical Research Centre, Cambridge Institute for Medical Research, and [3]Department of Biochemistry, University of Cambridge, Cambridge, United Kingdom

ABSTRACT FRET is widely used for the study of protein-protein interactions in biological samples. However, it is difficult to quantify both the FRET efficiency (E) and the affinity (K_d) of the molecular interaction from intermolecular FRET signals in samples of unknown stoichiometry. Here, we present a method for the simultaneous quantification of the complete set of interaction parameters, including fractions of bound donors and acceptors, local protein concentrations, and dissociation constants, in each image pixel. The method makes use of fluorescence lifetime information from both donor and acceptor molecules and takes advantage of the linear properties of the phasor plot approach. We demonstrate the capability of our method in vitro in a microfluidic device and also in cells, via the determination of the binding affinity between tagged versions of glutathione and glutathione S-transferase, and via the determination of competitor concentration. The potential of the method is explored with simulations.

Received for publication 2 October 2014 and in final form 9 January 2015.

*Correspondence: cfk23@cam.ac.uk

This is an open access article under the CC BY license (http://creativecommons.org/licenses/by/4.0/).

Edward Avezov and Simon C. Schlachter contributed equally to this work.

Förster resonance energy transfer (FRET) is widely applied in the study of molecular interactions and conformational changes in biological systems (1). Both the FRET efficiency, E, and the fraction of molecules participating in the interaction, f, are important parameters in biochemical research. A number of intensity-based FRET methods have been developed to quantify E and f (2,3). Those can be performed with basic fluorescence equipment, which is advantageous; but they also require extensive calibration protocols, which may lead to large cumulative errors.

Fluorescence lifetime imaging microscopy (FLIM) provides a more robust means of quantifying FRET interactions because the fluorescence lifetime is an inherently ratiometric measurement (4–6). In existing FLIM methods, the fluorescence decay can be analyzed either by decay-curve fitting (7) or by using a geometric global analysis approach, called the AB- (8,9) or phasor-plot method (10–14). Both FRET efficiency and molecular fractions of active donors (i.e., donors participating in the FRET process), f_D^{FRET}, can be recovered. The value f_D^{FRET} depends on several factors, such as local concentrations of donor and acceptor and the binding affinity between them. All of these are of interest, but they cannot be quantified without knowledge of the bound acceptor fraction (fraction of acceptors that are in complex with their binding partners), which is not traditionally available when only donor lifetimes are measured. Spectrally resolved FLIM has been applied for FRET measurements to improve both the separation of multiple lifetime components and the accuracy of recovered FRET efficiencies (6,15–17), but they have not been extended to the recovery of the acceptor stoichiometry. The lifetime ingrowth of acceptors has been exploited for the analysis of FRET stoichiometry (18,19); however, these methods are impractical when fluorescence bleedthrough from donor fluorophores contaminates the FRET signal, a problem for most FRET pairs, because then the bound acceptor fraction becomes difficult to retrieve.

Here, we present a method, which combines the advantages of FLIM and phasor plot techniques, taking into full account the presence of cross-excitation (direct excitation of the acceptor upon donor excitation) and donor fluorescence bleedthrough in the acceptor emission channel. FRET efficiency and molecular fractions of both the bound donor and acceptor molecules are recovered, as well as the dissociation constant K_d. Measurements in a maximum of only three spectral channels are required by our method, which we refer to as multichannel FLIM-FRET (MC-FLIM-FRET). The validity and potential of the method are explored with simulations, and demonstrated experimentally with

Editor: Paul Wiseman.
© 2015 The Authors
http://dx.doi.org/10.1016/j.bpj.2015.01.012

CrossMark

time-correlated single photon counting measurements in microfluidic devices and in cells. We quantified the binding affinity between glutathione (GSH) fused to the fluorescein derivative Oregon green (OG-GSH, donor) and glutathione S-transferase (GST) fused to the fluorescent protein mCherry (mCherry-GST, acceptor) for various protein stoichiometries (for details on constructs, see Section S2 in the Supporting Material).

Fig. 1 *a* shows the principle of MC-FLIM-FRET. The method requires the measurement of fluorescence decays in both the donor channel (donor excitation/donor emission) and the FRET channel (donor excitation/acceptor emission). Here the case for fluorophores exhibiting monoexponential decays is discussed. Multiexponential decays are discussed in Section S1 in the Supporting Material. Fluorescence measured in the donor channel only contains a mixture of signal from donors participating in FRET (active donors) and those that do not (passive donors) (3,6), hence the corresponding mixed phasor ($\vec{\tau}_{DM}$) lies along the line joining the phasors of active ($\vec{\tau}_D^{\mathrm{FRET}}$) and passive donors ($\vec{\tau}_D$). From the positions of $\vec{\tau}_{DM}$, both the active donor fractions, $f_D{}^{\mathrm{FRET}}$, and FRET efficiency, E, can be recovered as previously demonstrated ((10,11), and see Section S1 in the Supporting Material). The bound donor fraction f_D* is the same as $f_D{}^{\mathrm{FRET}}$ (see Section S1 in the Supporting Material for detail).

On the other hand, the phasor for the FRET channel, $\vec{\tau}_{DA}$, is a linear combination of active and passive acceptor phasors ($\vec{\tau}_A^{\mathrm{FRET}}$ and $\vec{\tau}_A$, respectively) and $\vec{\tau}_{DM}$ (combination of $\vec{\tau}_D^{\mathrm{FRET}}$ and $\vec{\tau}_D$) resulting from donor bleedthrough. The value $\vec{\tau}_A$ is easily obtained from a FLIM measurement in the acceptor channel (acceptor excitation/acceptor emission) using a sufficiently long excitation wavelength, or, alternatively, via measurement in a control sample containing acceptors only. The value $\vec{\tau}_A^{\mathrm{FRET}}$ is calculated from $\vec{\tau}_A$ and $\vec{\tau}_D^{\mathrm{FRET}}$ by considering the acceptor lifetime ingrowth using the methods detailed in Section S1 in the Supporting Material. The phasor $\vec{\tau}_{AM}$ (containing only the contribution from acceptors) can then be obtained by the intersection of the line $\vec{\tau}_{DM} - \vec{\tau}_{DA}$ (*blue line* in Fig. 1) with the acceptor phasor trajectory (*red line*), from which the fraction of FRET active acceptors $f_A{}^{\mathrm{FRET}}$ can finally be determined. Note that, due to cross-excitation, not all of the acceptor molecules bound to donor molecules are FRET-active. The fraction of bound acceptors f_A* can be recovered with $f_A{}^{\mathrm{FRET}}$ using the methods described in Section S1 in the Supporting Material. Hence, both FRET efficiency and stoichiometry are resolved with our method. If either donor concentration ([D]) or acceptor concentration ([A]) is known a priori, then K_d can also be recovered. If this is not the case, then [A] can be recovered from an intensity measurement in the acceptor channel. (See Section S1 in the Supporting Material for further explanations.)

To explore the dynamic range of MC-FLIM-FRET, we performed simulations using spectral parameters mimicking the OG/mCherry pair and eGFP/mCherry pair (see Section S4 in the Supporting Material for details). The simulations were performed in the presence of realistic levels of noise, and verify that donor- and acceptor-bound fractions as well as K_d can be recovered with good accuracy from data with signal levels typically available in real experiments.

Next, we validated the method experimentally by imaging a microfluidic device filled with ~500 microdroplets of

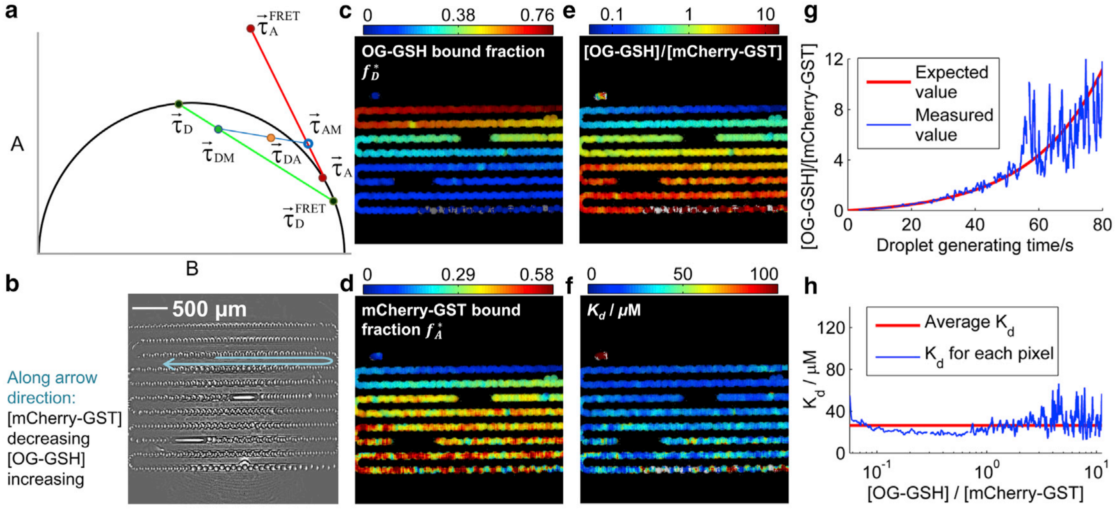

FIGURE 1 Principle of MC-FLIM-FRET, and validation. (*a*) Explanation of phasor plot construction for MC-FLIM-FRET. (*b*) Transmitted light image of a microfluidic device containing a sequence of microdroplets with continuously varying stoichiometry. (*c*) Recovered fraction of bound donor. (*d*) Recovered fraction of bound acceptor. (*e*) Recovered concentration ratio between donor and acceptor (log scale). (*f*) Recovered dissociation constant K_d. (*g*) Recovered concentration ratio between donor and acceptor (*blue line*), and expected value calculated from known mixing conditions during droplet generation. (*h*) The value K_d is verified to be independent of [D]/[A]. The average photon count in each binned pixel is ~14,000 for panels *c–f*, and ~90,000 for *g* and *h* (see Section S3 in the Supporting Material for details).

volume 3 nL, each containing a unique stoichiometry of OG-GSH and mCherry-GST with [D]/[A] ranging from 0.06 to 11.19 (see Section S2 in the Supporting Material for details). Fig. 1 *b* shows a transmitted light image of the microfluidic device. Fig. S13 *a* in the Supporting Material shows the corresponding phasor plot for the data from which a FRET efficiency of $E = (59.5 \pm 0.5)\%$ (errors quoted as SE, 68% confidence interval) is recovered. Fig. 1, *c* and *d*, presents the recovered bound fractions f_D* and f_A*, respectively, from which we obtain the [D]/[A] across the image (Fig. 1 *e*, and see Section S1 in the Supporting Material for details). Fig. 1 *f* shows the recovered dissociation constant K_d across the image. Because each spatial position inside the microfluidic device correlates with the time point when the mixture was generated, we can plot the temporal evolution of the concentration ratios measured (*blue curve*, Fig. 1 *g*) and compare this with the known values (*red curve*; see Section S2 in the Supporting Material for details). The data are in good agreement. Fig. 1 *h* shows the recovered K_d value for different donor and acceptor concentration ratios and as expected, the recovered K_d value is approximately constant throughout. We obtain a mean value of $K_d = 26.5 \pm 0.2$ μM. Fig. 1, *g* and *h*, shows how the sensitivity of the method decreases as [D]/[A] gets large, and signal/noise correspondingly small. In Section S4 in the Supporting Material we compare the experimental noise performance and measurement sensitivity with simulations, and both are in good agreement.

We also tested the performance of the method for measurements in cells, with autofluorescence taken into account (20). HEK293T cells expressing mCherry-GST were prepared and permeabilized with saponin, a mild detergent (21). OG-GSH was then added to the medium and its diffusion ensued into the cells. The cell-endogenous GSH, which is a competitor for the OG-GSH and mCherry-GST interaction, was depleted after membrane permeabilization (Section S2 in the Supporting Material). Fig. 2, *a* and *b*, shows the bound fractions of donors and acceptors, respectively, for a representative cell. The recovered FRET efficiency is $(58.7 \pm 0.6)\%$. Using a further measurement in the acceptor channel, we recovered the acceptor concentration [A] (see Section S3 in the Supporting Material), and hence, K_d, as shown in Fig. 2 *c*. We obtain an average value of $K_d = 37.2 \pm 0.2$ μM. Although similar to the microdroplet result, the difference is likely to reflect the residual presence of endogenous GSH and the different solution conditions prevailing in the cell. Next, we added 200 μM GSH to the medium to introduce the effect of a competitor. Fig. 2, *d* and *e*, shows the recovered f_D* and f_A* in this case; both are lower than in absence of competitor, as expected. The calculated apparent K_d, Fig. 2 *f*, is now clearly larger than in the GSH-depleted sample shown in Fig. 2 *c*. Assuming that the real K_d value is unchanged, we can now recover the concentration of the competitor, GSH (Fig. 2 *g*). We thus obtain a GSH concentration of 93.3 ± 0.3 μM. This reduced concentration is likely reflective of

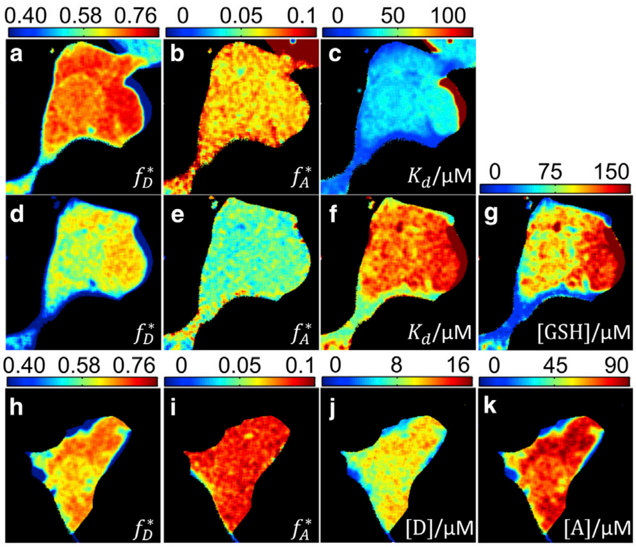

FIGURE 2 Validation of MC-FLIM-FRET in cells. (*a–c*) Bound fractions and dissociation constants. (*d–g*) Recovered parameters upon adding competitor GSH. (*h–k*) Absolute concentration determination in cells with known $K_d = 37.2 \pm 0.2$ μM. The average photon count in each binned pixel is ~13,000 (see Section S3 in the Supporting Material for details).

the fact that GSH undergoes oxidation during sample preparation (Section S2 in the Supporting Material). Finally, even in the case where neither donor nor acceptor concentrations are available, it is possible to recover variations in K_d and competitor concentrations across a sample (see Section S1 in the Supporting Material for details).

On the other hand, for a known K_d value in a bimolecular complex, and in the absence of competitor reactions, both absolute donor and acceptor concentrations can be recovered (Section S1 in the Supporting Material). In Fig. 2, *h* and *i*, the bound fractions f_D* and f_A* are presented for another cell. Assuming a K_d value as was measured in Fig. 2 *c*, [D] and [A] can be recovered in the cell (Fig. 2, *j* and *k*). The average [A] recovered in this way is (66.9 ± 0.2) μM, which compares well with an acceptor-intensity-based measurement of (50.8 ± 0.1) μM, giving confidence to both the robustness of the method and the extracted value for K_d.

In summary, we have developed a robust method for FRET quantification using FLIM measurements in both the donor and acceptor emission channels, in combination with a powerful phasor plot approach. It permits us to compensate for donor bleedthrough and acceptor cross-excitation, recovering both FRET efficiency and molecular fractions of bound donor and acceptor complexes, unachievable with common FLIM-FRET techniques. The method was validated using simulation, microfluidic experiments, and cell experiments. Our method is useful for measurements of dissociation constants, donor and acceptor concentrations, and the presence and concentration of competitors to binding reactions.

SUPPORTING MATERIAL

Supporting Materials and Methods, 13 figures, and one table are available at http://www.biophysj.org/biophysj/supplemental/S0006-3495(15)00075-2.

AUTHOR CONTRIBUTIONS

W.Y.C. designed and performed the research, analyzed the data, and wrote the manuscript. E.A. contributed tools and performed the research. S.C.S. contributed analytic tools and performed the research. F.G. contributed tools and performed the research. R.F.L. analyzed the data. H.P.H. performed the research. F.H. contributed tools. D.R. designed the research and contributed tools. C.F.K. designed the research, supervised the project, and wrote the manuscript.

ACKNOWLEDGMENTS

MATLAB (The MathWorks, Natick, MA) codes for MC-FLIM-FRET analyses are available at laser.ceb.cam.ac.uk.

We thank Ana Crespillo-Casada for sample purifications and Dr. Angus Bain for useful discussions.

This work was funded by grants from the Medical Research Council, the Wellcome Trust, the Alzheimer Research UK Trust, and the Engineering and Physical Sciences Research Council. W.Y.C. is funded by a China Scholarship Council-Cambridge Scholarship. D.R. is a Principal Research Fellow of the Wellcome Trust.

SUPPORTING CITATIONS

References (22–32) appear in the Supporting Material.

REFERENCES

1. Selvin, P. R. 2000. The renaissance of fluorescence resonance energy transfer. *Nat. Struct. Biol.* 7:730–734.
2. Hoppe, A., K. Christensen, and J. A. Swanson. 2002. Fluorescence resonance energy transfer-based stoichiometry in living cells. *Biophys. J.* 83:3652–3664.
3. Elder, A., A. Domin, …, C. Kaminski. 2009. A quantitative protocol for dynamic measurements of protein interactions by Förster resonance energy transfer-sensitized fluorescence emission. *J. R. Soc. Interface.* 6:S59–S81.
4. Lakowicz, J. R. 2006. Principles of Fluorescence Spectroscopy. Springer, New York.
5. Padilla-Parra, S., and M. Tramier. 2012. FRET microscopy in the living cell: different approaches, strengths and weaknesses. *BioEssays.* 34:369–376.
6. Biskup, C., T. Zimmer, …, K. Benndorf. 2007. Multi-dimensional fluorescence lifetime and FRET measurements. *Microsc. Res. Tech.* 70:442–451.
7. Millington, M., G. J. Grindlay, …, S. W. Magennis. 2007. High-precision FLIM-FRET in fixed and living cells reveals heterogeneity in a simple CFP-YFP fusion protein. *Biophys. Chem.* 127:155–164.
8. Clayton, A. H., Q. S. Hanley, and P. J. Verveer. 2004. Graphical representation and multicomponent analysis of single-frequency fluorescence lifetime imaging microscopy data. *J. Microsc.* 213:1–5.
9. Forde, T. S., and Q. S. Hanley. 2006. Spectrally resolved frequency domain analysis of multi-fluorophore systems undergoing energy transfer. *Appl. Spectrosc.* 60:1442–1452.
10. Digman, M. A., V. R. Caiolfa, …, E. Gratton. 2008. The phasor approach to fluorescence lifetime imaging analysis. *Biophys. J.* 94:L14–L16.
11. Laine, R., D. W. Stuckey, …, P. M. French. 2012. Fluorescence lifetime readouts of Troponin-C-based calcium FRET sensors: a quantitative comparison of CFP and mTFP1 as donor fluorophores. *PLoS ONE.* 7:e49200.
12. Redford, G. I., and R. M. Clegg. 2005. Polar plot representation for frequency-domain analysis of fluorescence lifetimes. *J. Fluoresc.* 15: 805–815.
13. Štefl, M., N. G. James, …, D. M. Jameson. 2011. Applications of phasors to in vitro time-resolved fluorescence measurements. *Anal. Biochem.* 410:62–69.
14. Hinde, E., M. A. Digman, …, E. Gratton. 2012. Biosensor Förster resonance energy transfer detection by the phasor approach to fluorescence lifetime imaging microscopy. *Microsc. Res. Tech.* 75:271–281.
15. Strat, D., F. Dolp, …, A. Rueck. 2011. Spectrally resolved fluorescence lifetime imaging microscopy: Förster resonant energy transfer global analysis with a one- and two-exponential donor model. *J. Biomed. Opt.* 16:026002.
16. Chen, Y. C., and R. M. Clegg. 2011. Spectral resolution in conjunction with polar plots improves the accuracy and reliability of FLIM measurements and estimates of FRET efficiency. *J. Microsc.* 244:21–37.
17. Fereidouni, F., G. A. Blab, and H. C. Gerritsen. 2014. Phasor based analysis of FRET images recorded using spectrally resolved lifetime imaging. *Methods Appl. Fluoresc.* 2:035001.
18. Laptenok, S. P., J. W. Borst, …, H. van Amerongen. 2010. Global analysis of Förster resonance energy transfer in live cells measured by fluorescence lifetime imaging microscopy exploiting the rise time of acceptor fluorescence. *Phys. Chem. Chem. Phys.* 12:7593–7602.
19. Visser, N. V., J. W. Borst, …, A. J. Visser. 2005. Direct observation of resonance tryptophan-to-chromophore energy transfer in visible fluorescent proteins. *Biophys. Chem.* 116:207–212.
20. Szmacinski, H., V. Toshchakov, and J. R. Lakowicz. 2014. Application of phasor plot and autofluorescence correction for study of heterogeneous cell population. *J. Biomed. Opt.* 19:046017.
21. Medepalli, K., B. W. Alphenaar, …, P. Sethu. 2013. A new technique for reversible permeabilization of live cells for intracellular delivery of quantum dots. *Nanotechnology.* 24:205101.
22. Colyer, R. A., O. H. Siegmund, …, X. Michalet. 2012. Phasor imaging with a widefield photon-counting detector. *J. Biomed. Opt.* 17:016008.
23. Kaminski, C. F., E. J. Rees, and G. S. K. Schierle. 2014. A quantitative protocol for intensity-based live cell FRET imaging. *In* Fluorescence Spectroscopy and Microscopy. Humana Press, Totowa, NY, pp. 445–454.
24. Schlachter, S., A. D. Elder, …, C. F. Kaminski. 2009. mhFLIM: resolution of heterogeneous fluorescence decays in widefield lifetime microscopy. *Opt. Express.* 17:1557–1570.
25. Martynov, V. I., B. I. Maksimov, …, S. A. Lukyanov. 2003. A purple-blue chromoprotein from *Goniopora tenuidens* belongs to the DsRed subfamily of GFP-like proteins. *J. Biol. Chem.* 278:46288–46292.
26. Zagranichny, V. E., N. V. Rudenko, …, A. S. Arseniev. 2004. zFP538, a yellow fluorescent protein from coral, belongs to the DsRed subfamily of GFP-like proteins but possesses the unexpected site of fragmentation. *Biochemistry.* 43:4764–4772.
27. Xia, Y., and G. M. Whitesides. 1998. Soft lithography. *Annu. Rev. Mater. Sci.* 28:153–184.
28. Devenish, S. R., M. Kaltenbach, …, F. Hollfelder. 2013. Droplets as reaction compartments for protein nanotechnology. *In* Protein Nanotechnology. Springer, New York, pp. 269–286.
29. Gielen, F., T. Buryska, …, F. Hollfelder. 2015. Interfacing microwells with nanoliter compartments: a sampler generating high-resolution concentration gradients for quantitative biochemical analyses in droplets. *Anal. Chem.* 87:624–632.
30. Chan, F. T., G. S. Kaminski Schierle, …, C. F. Kaminski. 2013. Protein amyloids develop an intrinsic fluorescence signature during aggregation. *Analyst (Lond.).* 138:2156–2162.
31. Wahl, M. 2014. Time-Correlated Single Photon Counting. (Technical report). PicoQuant, Berlin, Germany.
32. Leray, A., C. Spriet, …, L. Héliot. 2012. Generalization of the polar representation in time domain fluorescence lifetime imaging microscopy for biological applications: practical implementation. *J. Microsc.* 248:66–76.

A Method to Quantify FRET Stoichiometry with Phasor Plot Analysis and Acceptor Lifetime In-growth

WeiYue Chen,* Edward Avezov,[†%] Simon C. Schlachter, *[%] Fabrice Gielen, [‡] Romain F. Laine,* Heather P. Harding,[†] Florian Hollfelder, [‡] David Ron,[†] and Clemens F. Kaminski*[&]

*Department of Chemical Engineering and Biotechnology, University of Cambridge, Cambridge, UK; [†] The Wellcome Trust MRC Institute of Metabolic Science and NIHR Cambridge Biomedical Research Centre, Cambridge Institute for Medical Research, University of Cambridge, Cambridge, UK; [‡] Department of Biochemistry, University of Cambridge, Cambridge, UK; [%]Contributed equally.

Supporting Material

S1. Theory

1) Phasor plot

For the analysis of time-domain FLIM data using the phasor plot (1), the time-dependent fluorescence intensity *I(t)* is measured in each pixel and its normalized Fourier transform is calculated at the first harmonic of the laser repetition rate (angular frequency ω). This yields a complex number which represents a vector in the complex plane, the so called “phasor” denoted by $\vec{\tau}$ in Eq. S1.

$$\vec{\tau} = \mathcal{F}\left(I(t)\right) = \frac{\int_0^\infty I(t)\mathrm{e}^{i\omega t}\,\mathrm{d}t}{\int_0^\infty I(t)\,\mathrm{d}t} = Ai + B = \overbrace{\frac{\int_0^\infty I(t)\sin(\omega t)\,\mathrm{d}t}{\int_0^\infty I(t)\,\mathrm{d}t}}^{A} i + \overbrace{\frac{\int_0^\infty I(t)\cos(\omega t)\,\mathrm{d}t}{\int_0^\infty I(t)\,\mathrm{d}t}}^{B} \qquad \text{S1}$$

In Eq. S1, *A* and *B* are the phasor coordinates representing the imaginary and real parts of the Fourier transform respectively. Frequency domain FLIM, FD-FLIM, is an alternative method of measuring fluorescence lifetimes and will not be treated here, but data obtained using that method can be directly represented on a phasor plot and the unmixing algorithms derived later applies identically to the recovery of FRET stoichiometries. For use of phasors in FD-FLIM the reader is referred to Digman *et al.* (1).

For a single exponential intensity decay

$$I(t) = I_0 e^{-t/\tau} \qquad \text{S2}$$

We can derive the coordinates *A* and *B* of the phasor using Eq. S3, obtained directly from Eq. S1:

$$A = \frac{\omega\tau}{(\omega\tau)^2 + 1},\ \ B = \frac{1}{(\omega\tau)^2 + 1} \qquad \text{S3}$$

Therefore, the phasors of single-exponential intensity decays define the so-called ‘universal circle’, as described by Eq. S4:

$$A^2 + (B - 1/2)^2 = 1/4 \qquad \text{S4}$$

As shown previously (1), the addition of phasors obeys the vector addition rules. For a sample containing several single-exponential decay components, the fluorescence intensity decay can be expressed as:

$$I(t) = I_0 \sum_n (f_n\, \mathrm{e}^{-t/\tau_n}) \qquad \text{S5}$$

where I_0 is the intensity at *t*=0, and f_n the fractional contribution of the corresponding exponential decay with lifetime τ_n. The phasor for such a multi-exponential decay can be calculated from Eq. S1 as:

$$\vec{\tau} = \frac{\sum_n f_n \tau_n \vec{\tau}_n}{\sum_n f_n \tau_n} = \frac{\sum_n I_n \vec{\tau}_n}{\sum_n I_n} = Ai + B = \overbrace{\frac{\sum_n I_n A_n}{\sum_n I_n}}^{A} i + \overbrace{\frac{\sum_n I_n B_n}{\sum_n I_n}}^{B} \qquad \text{S6}$$

where A_n and B_n are the phasor coordinates of the nth exponential decay component and I_n its corresponding steady-state fluorescence intensity. Therefore, if several signals contribute to a pixel, the phasor of that pixel can be obtained by the sum of the phasors of all signal components that contribute to the pixel, weighted by their intensity fraction. As a consequence, the phasor of a multi-exponential intensity decay falls inside the

universal circle. In a spatially heterogeneous sample where different pixels contain different fractional intensity contributions from the same individual decay components, it is possible to retrieve individual lifetime components using the rules of vector addition and this forms the essence what is called a 'global analysis'. For the particular case where only two components contribute, the phasors will lie on a straight line between the positions corresponding of the two components. For more details the reader is referred to references (1, 2).

2) Theoretical derivation for Acceptor FLIM-FRET method

The time evolution of the excited-state population N of an ensemble of molecules can be obtained from:

$$\frac{\mathrm{d}N}{\mathrm{d}t} = -\sum_i k_i N \qquad \text{S7}$$

where k_i denotes the decay rate (in second^{-1}) of different decay paths that the molecule can undergo.

Comparing Eq. S7 with Eq. S2, we recognize the lifetime of the molecule to be

$$\tau = \frac{1}{\sum_i k_i} \qquad \text{S8}$$

Here, donors that bind to the acceptors are referred to as "bound donors", while those donors not binding to the acceptors are referred to as "unbound donors". The donors that undergo FRET are referred to as "active donors", while those which are not as "passive donors". The same nomenclature is used in what follows for acceptors.

We note that for the donor molecules, all bound donors are active and all unbound donors are passive so the distinction is redundant. However, when considering the fluorescence decays from the acceptor, it is important to note that all unbound acceptors are passive (not undergoing FRET) but, not all bound acceptors are active (undergoing FRET). From the bound population of acceptor, a fraction is active and a fraction is passive, hence the need for this classification.

Although the method that we describe in this article is based on the assumption that the donor and acceptor fluorescence decays are approximately mono-exponential, it can be extended to cases when donor and acceptor have multi-exponential decays if respective components are known. In what follows we treat first the case when both the donor and acceptor fluorescence decays exhibit mono-exponential lifetime decays. We will investigate the case for multi-exponential donor and acceptor lifetime decays in the following section.

Description of the method for mono-exponential donor and acceptor

For unbound donor and unbound acceptor, no FRET occurs for them. The time evolutions of the excited-state population of unbound donors and unbound acceptors are described as follows:

$$\begin{cases} \dfrac{\mathrm{d}N_D(t)}{\mathrm{d}t} = -\left(k_{rD} + k_{nrD}\right) N_D(t) = -\dfrac{1}{\tau_D} N_D(t) \\ \dfrac{\mathrm{d}N_A(t)}{\mathrm{d}t} = -\left(k_{rA} + k_{nrA}\right) N_A(t) = -\dfrac{1}{\tau_A} N_A(t) \end{cases} \qquad \text{S9}$$

Here $N_D(t)$ and $N_A(t)$ are the excited-state populations of unbound donor and unbound acceptor respectively. k_{rD} and k_{rA} represent their respective radiative decay rates (Einstein coefficient for spontaneous emission). k_{nrD} and k_{nrA} denote corresponding non-radiative decay rates. $\tau_D = 1/(k_{rD} + k_{nrD})$ and $\tau_A = 1/(k_{rA} + k_{nrA})$ thus represent the lifetimes of passive donors and passive acceptors, respectively.

The time evolution of the excited-state populations $N_D^*(t)$ and $N_A^*(t)$ for bound donors and bound acceptors can be written as follows (also see (3) for details):

$$\begin{cases} \dfrac{\mathrm{d}N_D^*(t)}{\mathrm{d}t} = -\left(k_{rD} + k_{nrD} + k_{FRET}\right) N_D^*(t) = -\dfrac{1}{\tau_D^{FRET}} N_D^*(t) \\ \dfrac{\mathrm{d}N_A^*(t)}{\mathrm{d}t} = k_{FRET} N_D^*(t) - \left(k_{rA} + k_{nrA}\right) N_A^*(t) = \dfrac{1}{\tau_D^{FRET}} E N_D^*(t) - \dfrac{1}{\tau_A} N_A^*(t) \end{cases} \qquad \text{S10}$$

Here k_{FRET} represents the FRET transfer rate, $\tau_D^{FRET} = 1/(k_{rD} + k_{nrD} + k_{FRET})$ is the lifetime of FRET active donors. E denotes the FRET efficiency between the active donors and acceptors, also referred to as the FRET quantum yield (4):

$$E = \frac{k_{FRET}}{k_{rD} + k_{nrD} + k_{FRET}} \quad \text{S11}$$

Hence:

$$E = 1 - \frac{\tau_D^{FRET}}{\tau_D} \quad \text{S12}$$

The 'source term' $k_{FRET} N_D^*(\mathrm{t})$ in Eq. S10 quantifies the energy transfer from the donor to the acceptor *via* FRET, and is the cause for the temporal 'in-growth' (4) in the active acceptor intensity decay.

In practice, one excites the sample with a short light pulse over a narrow bandwidth of the donor excitation spectrum. The probabilities that the donor and acceptor undergo transitions to the excited-states are $\mathcal{B}_D^{Dex} P^{Dex}$ and $\mathcal{B}_A^{Dex} P^{Dex}$ respectively, where $\mathcal{B}_D^{Dex}$ and $\mathcal{B}_A^{Dex}$ are the Einstein coefficients for photon absorption by the donor and acceptor at donor excitation wavelength, and P^{Dex} is the corresponding excitation power. The initial conditions for Eq. S9 and S10 are therefore given by :

$$\begin{cases} N_D(0) = n_D \mathcal{B}_D^{Dex} P^{Dex} \\ N_D^*(0) = n_D^* \mathcal{B}_D^{Dex} P^{Dex} \\ N_A(0) = n_A \mathcal{B}_A^{Dex} P^{Dex} \\ N_A^*(0) = n_A^* \mathcal{B}_A^{Dex} P^{Dex} \end{cases} \quad \text{S13}$$

where n_D, n_D^*, n_A and n_A^* represent the total number of unbound donors, bound donors, unbound acceptors and bound acceptors, respectively, in the system. Note that for a 1:1 interaction, $n_D^* = n_A^*$.

By solving Eq. S9, S10 and S13, the time-evolution of the excited-state population of donor and acceptor after donor excitation is obtained:

$$\begin{cases} N_D^{tot}(t) = \overbrace{\mathcal{B}_D^{Dex} P^{Dex} n_D^* e^{-t/\tau_D^{FRET}}}^{N_D^*(t)} + \overbrace{\mathcal{B}_D^{Dex} P^{Dex} n_D e^{-t/\tau_D}}^{N_D(t)} \\ \begin{cases} N_A^{tot}(t) = \overbrace{\mathcal{B}_D^{Dex} P^{Dex} n_D^* E \frac{\tau_A}{\tau_A - \tau_D^{FRET}} (e^{-t/\tau_A} - e^{-t/\tau_D^{FRET}}) + \mathcal{B}_A^{Dex} P^{Dex} n_A^* e^{-t/\tau_A}}^{N_A^*(\mathrm{t})} + \overbrace{\mathcal{B}_A^{Dex} P^{Dex} n_A e^{-t/\tau_A}}^{N_A(\mathrm{t})} & (\text{if } \tau_A \neq \tau_D^{FRET}) \\ N_A^{tot}(t) = \overbrace{\mathcal{B}_D^{Dex} P^{Dex} n_D^* E \frac{t}{\tau_A} e^{-t/\tau_A} + \mathcal{B}_A^{Dex} P^{Dex} n_A^* e^{-t/\tau_A}}^{N_A^*(\mathrm{t})} + \overbrace{\mathcal{B}_A^{Dex} P^{Dex} n_A e^{-t/\tau_A}}^{N_A(\mathrm{t})} & (\text{if } \tau_A = \tau_D^{FRET}) \end{cases} \end{cases} \quad \text{S14}$$

Here $N_D^{tot}(t)$ and $N_A^{tot}(t)$ are the excited-state populations, respectively, of the total all donors and all acceptors. Note that in most cases $\tau_A \neq \tau_D^{FRET}$.

Equation S14 shows that the time-evolution of the excited-state population of bound acceptors:

$$N_A^*(t) = \overbrace{\mathcal{B}_D^{Dex} P^{Dex} n_D^* E \frac{\tau_A}{\tau_A - \tau_D^{FRET}} (e^{-t/\tau_A} - e^{-t/\tau_D^{FRET}})}^{Undergoing\ FRET} + \overbrace{\mathcal{B}_A^{Dex} P^{Dex} n_A^* e^{-t/\tau_A}}^{not\ undergoing\ FRET} \quad \text{S15}$$

The first part contains the in-growth effect due to energy transfer from the donor and the decay term corresponding to the acceptor molecules that were excited *via* FRET. This term therefore represents the bound acceptors that undergo FRET. The second part corresponds to the fluorescence decay associated with the passive acceptors. It represents those acceptors which are bound but are not undergoing FRET. The existence of bound, but FRET passive, acceptors is a manifestation of direct excitation of some bound acceptors at the donor excitation wavelength. This term is unwanted as it does not correspond to a FRET signal. In practice, this term can be minimized but it is very challenging to exclude it totally from the measurement with typical FRET pairs (small Stokes shift) and therefore, $N_A^*(0)$ in Eq. S13 is typically non-zero. In the literature, this contribution is often referred to as cross-excitation (5, 6). To the observer, the fluorescence properties of acceptors thus excited is indistinguishable to those of unbound acceptors; they are passive acceptors (see also Fig. S1 for illustration of that notion).

For donors, on the other hand, we assume that all bound donors are FRET active, as described in Eq. S10. Theoretically, it is possible that both donor and acceptor in a bound complex are simultaneously excited by the laser pulse, thus preventing FRET from occurring. In practice, the probability for this to occur is negligible for several reasons: 1) In TCSPC based experiments, laser powers are very low and only a small portion of donors and

acceptors are excited per pulse. It is thus very unlikely to excite both donor and acceptor in the same bound pair simultaneously. 2) The probability of direct excitation of bound acceptors is much lower than that of direct excitation of bound donors at the donor excitation wavelength.

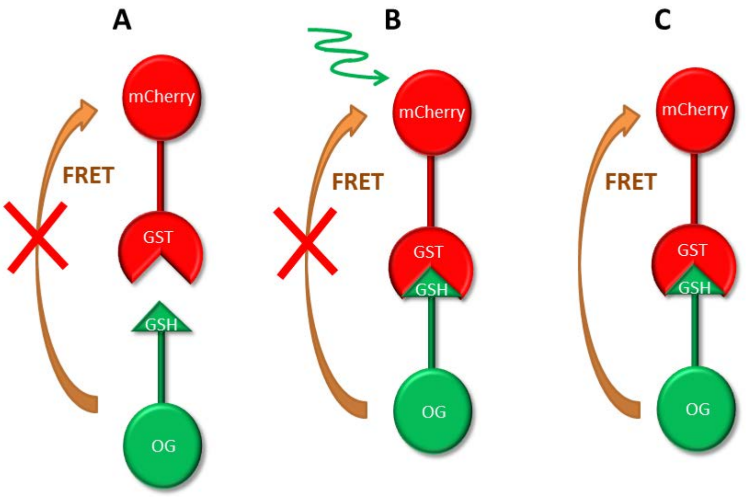

Figure S1. Illustration of the terminologies for acceptors used throughout paper. Green arrow: excitation photon at donor excitation wavelength. Brown arrow: FRET path. A: Unbound and passive acceptor. B: bound but passive acceptor due to cross-excitation. C: bound and active acceptor.

The time-dependent intensity (number of photons emitted) from the donor channel ($I_D^{tot}(t)$) and from the acceptor channel ($I_A^{tot}(t)$) can then be written as follows.

$$\begin{cases} I_D^{tot}(t) = N_D^*(t)k_{rD} + N_D(t)k_{rD} \\ I_A^{tot}(t) = N_A^*(t)k_{rA} + N_A(t)k_{rA} \end{cases} \qquad \text{S16}$$

However, experimentally, donor bleed-through (donor fluorescence signal leaking into acceptor detection channel) and acceptor cross-excitation (direct excitation of acceptors upon donor excitation) need to be taken into account. Therefore, the fluorescence intensity measured in the donor emission channel upon excitation at the donor wavelength ($I^{DexDem}(t)$), that measured in the acceptor emission channel upon excitation at the donor wavelength ($I^{DexAem}(t)$) and that measured in the acceptor emission channel upon excitation at the acceptor wavelength ($I^{AexAem}(t)$) can be expressed as follows:

$$\begin{cases} I^{AexAem}(t) = \mathcal{B}_A^{Aex} P^{Aex}(n_A + n_A^*)e^{-t/\tau_A} k_{rA}\psi_A^{Aem} \\ I^{DexDem}(t) = \mathcal{B}_D^{Dex} P^{Dex}(n_D e^{-t/\tau_D} + n_D^* e^{-t/\tau_D^{FRET}})k_{rD}\psi_D^{Dem} \\ I^{DexAem}(t) = \overbrace{\mathcal{B}_D^{Dex} P^{Dex} n_D^* E \frac{\tau_A}{\tau_A - \tau_D^{FRET}}(e^{-t/\tau_A} - e^{-t/\tau_D^{FRET}})k_{rA}\psi_A^{Aem} + \mathcal{B}_A^{Dex} P^{Dex} n_A^* e^{-t/\tau_A} k_{rA}\psi_A^{Aem}}^{\text{from interacting acceptors}} \\ \qquad + \overbrace{\mathcal{B}_A^{Dex} P^{Dex} n_A e^{-t/\tau_A} k_{rA}\psi_A^{Aem}}^{\text{from non-interacting acceptors}} \\ \qquad + \overbrace{\mathcal{B}_D^{Dex} P^{Dex} n_D^* e^{-t/\tau_D^{FRET}} k_{rD}\psi_D^{Aem}}^{\text{from interacting donors}} + \overbrace{\mathcal{B}_D^{Dex} P^{Dex} n_D e^{-t/\tau_D} k_{rD}\psi_D^{Aem}}^{\text{from non-interacting donors}} \end{cases} \qquad \text{S17}$$

Here $\mathcal{B}_A^{Aex}$ is the Einstein coefficient for photon absorption by the acceptor at acceptor excitation wavelength, and P^{Aex} is the corresponding excitation power. ψ represents the photon detection efficiency, *i.e.* ψ_D^{Dem} represents the fraction of donor photons collected by the detector in the donor emission channel, ψ_D^{Aem} the fraction of donor photons collected by the detector in the acceptor emission channel, and ψ_A^{Aem} the fraction of acceptor photons detected by the detector in the acceptor emission channel.

From Eq. S1, Eq. S6 and Eq. S17, we can derive an expression for the phasor obtained for the signal measured in the donor channel (donor excitation / donor emission), as follows:

$$\vec{\tau}_{DM} = \frac{n_D \tau_D \vec{\tau}_D + n_D^* \tau_D^{FRET} \vec{\tau}_D^{FRET}}{n_D \tau_D + n_D^* \tau_D^{FRET}} = \frac{I_D \vec{\tau}_D + I_D^{FRET} \vec{\tau}_D^{FRET}}{I_D + I_D^{FRET}} \qquad \text{S18}$$

where $\vec{\tau}_D$ and $\vec{\tau}_D^{FRET}$ the phasors of passive and active donors, I_D and I_D^{FRET} the steady-state fluorescence intensity of passive and active donor. From the properties of vector addition of the phasor representation, we know that $\vec{\tau}_{DM}$ lies on the line joining $\vec{\tau}_D$ and $\vec{\tau}_D^{FRET}$. Therefore, if the donor exhibits a single-exponential decay, $\vec{\tau}_D$ and $\vec{\tau}_D^{FRET}$ can be obtained by cutting the $\vec{\tau}_{DM}$ trajectory with the universal circle.

The phasor of the active acceptor ($\vec{\tau}_A^{FRET}$) and the corresponding coordinates (A_A^{FRET} and B_A^{FRET}) can be calculated from Eq. S1, Eq. S15 and Eq. S17, leading to:

$$\vec{\tau}_A^{FRET} = \frac{\int_0^\infty (e^{-t/\tau_A} - e^{-t/\tau_D^{FRET}}) e^{i\omega t}\,\mathrm{d}t}{\int_0^\infty (e^{-t/\tau_A} - e^{-t/\tau_D^{FRET}})\,\mathrm{d}t} = \frac{\tau_A \vec{\tau}_A - \tau_D^{FRET} \vec{\tau}_D^{FRET}}{\tau_A - \tau_D^{FRET}} = i\overbrace{\frac{\tau_A A_A - \tau_D^{FRET} A_D^{FRET}}{\tau_A - \tau_D^{FRET}}}^{A_A^{FRET}} + \overbrace{\frac{\tau_A B_A - \tau_D^{FRET} B_D^{FRET}}{\tau_A - \tau_D^{FRET}}}^{B_A^{FRET}} \qquad \text{S19}$$

where A_A and B_A are the phasor coordinates of the passive acceptor, A_D^{FRET} and B_D^{FRET} are the phasor coordinates of the active donor.

Similarly the phasor measured in the FRET channel (donor excitation / acceptor emission) representing fluorescence only from the acceptors ($\vec{\tau}_{AM}$) can be derived as follows:

$$\begin{aligned}\vec{\tau}_{AM} &= \mathcal{F}\Bigg(\overbrace{\mathcal{B}_D^{Dex} P^{Dex} n_D^* E \frac{\tau_A}{\tau_A - \tau_D^{FRET}} (e^{-t/\tau_A} - e^{-t/\tau_D^{FRET}}) k_{rA} \psi_A^{Aem} + \mathcal{B}_A^{Dex} P^{Dex} n_A^* e^{-t/\tau_A} k_{rA} \psi_A^{Aem}}^{\text{from interacting acceptors}} \\ &\quad + \overbrace{\mathcal{B}_A^{Dex} P^{Dex} n_A e^{-t/\tau_A} k_{rA} \psi_A^{Aem}}^{\text{from non-interacting acceptors}}\Bigg) \qquad \text{S20} \\ &= \frac{\mathcal{B}_D^{Dex} n_A^* E \vec{\tau}_A^{FRET} + \mathcal{B}_A^{Dex} (n_A^* + n_A) \vec{\tau}_A}{\mathcal{B}_D^{Dex} n_A^* E + \mathcal{B}_A^{Dex} (n_A^* + n_A)} = \frac{I_A^{FRET} \vec{\tau}_A^{FRET} + I_A \vec{\tau}_A}{I_A^{FRET} + I_A}\end{aligned}$$

where I_A^{FRET} and I_A are respectively the steady-state fluorescence intensity of the active and passive acceptors. Clearly $\vec{\tau}_{AM}$ lies on the line joining $\vec{\tau}_A^{FRET}$ and $\vec{\tau}_A$.

The phasor of total fluorescence measured in the FRET channel ($\vec{\tau}_{DA}$) can be calculated using Eq. S1 and Eq. S17:

$$\begin{aligned}\vec{\tau}_{DA} = \mathcal{F}\left(I^{DexAem}(t)\right) &= \frac{k_{rA}\psi_A^{Aem} \tau_A \left(\mathcal{B}_D^{Dex} n_D^* E \vec{\tau}_A^{FRET} + \mathcal{B}_A^{Dex} (n_A^* + \mathrm{n}_A) \vec{\tau}_A\right) + k_{rD} \psi_D^{Aem} \mathcal{B}_D^{Dex} (n_D \tau_D \vec{\tau}_D + n_D^* \tau_D^{FRET} \vec{\tau}_D^{FRET})}{k_{rA}\psi_A^{Aem} \tau_A \left(\mathcal{B}_D^{Dex} n_D^* E + \mathcal{B}_A^{Dex} (n_A^* + \mathrm{n}_A)\right) + k_{rD} \psi_D^{Aem} \mathcal{B}_D^{Dex} (n_D \tau_D + n_D^* \tau_D^{FRET})} \\ &= \frac{k_{rA}\psi_A^{Aem} \tau_A \left(\mathcal{B}_D^{Dex} n_D^* E + \mathcal{B}_A^{Dex} (n_A^* + n_A)\right) \vec{\tau}_{AM} + k_{rD} \psi_D^{Aem} \mathcal{B}_D^{Dex} (n_D \tau_D + n_D^* \tau_D^{FRET}) \vec{\tau}_{DM}}{k_{rA}\psi_A^{Aem} \tau_A \left(\mathcal{B}_D^{Dex} n_D^* E + \mathcal{B}_A^{Dex} (n_A^* + \mathrm{n}_A)\right) + k_{rD} \psi_D^{Aem} \mathcal{B}_D^{Dex} (n_D \tau_D + n_D^* \tau_D^{FRET})} \qquad \text{S21} \\ &= \frac{I_{AM} \vec{\tau}_{AM} + I_{DM} \vec{\tau}_{DM}}{I_{AM} + I_{DM}}\end{aligned}$$

Here I_{AM} is the steady-state fluorescence intensity of acceptors, and I_{DM} is the steady-state fluorescence intensity of donors. We therefore note that the phasor $\vec{\tau}_{DA}$ sits on the line joining $\vec{\tau}_{AM}$ and $\vec{\tau}_{DM}$. Therefore, with our method, the position of $\vec{\tau}_{AM}$ can be found from the intersection of the line joining $\vec{\tau}_{DM}$ and $\vec{\tau}_{DA}$ with that joining $\vec{\tau}_A$ and $\vec{\tau}_A^{FRET}$ as shown in Fig. 1a in the main artile.

Using Eq. S6 and Eq. S18, the intensity contribution from the fraction of active donors and active acceptors (called here α_D^{FRET} and α_A^{FRET}, respectively) can now be directly calculated:

$$\begin{cases} \alpha_D^{FRET} = \dfrac{I_D^{FRET}}{I_D^{FRET}+I_D} = \dfrac{\|\vec{\tau}_D - \vec{\tau}_{DM}\|}{\|\vec{\tau}_D - \vec{\tau}_D^{FRET}\|} \\ \alpha_A^{FRET} = \dfrac{I_A^{FRET}}{I_A^{FRET}+I_A} = \dfrac{\|\vec{\tau}_A - \vec{\tau}_{AM}\|}{\|\vec{\tau}_A - \vec{\tau}_A^{FRET}\|} \end{cases} \qquad \text{S22}$$

The molecular fraction of active donors and active acceptors can then be calculated from Eq. S18, Eq. S20 and Eq. S22:

$$\begin{cases} f_D^{FRET} = \dfrac{n_D^*}{n_D + n_D^*} = \dfrac{\alpha_D^{FRET}\tau_D}{(1-\alpha_D^{FRET})\tau_D^{FRET} + \alpha_D^{FRET}\tau_D} \\ f_A^{FRET} = \dfrac{n_A^*}{n_A^* + (n_A^* + n_A)} = \dfrac{\alpha_A^{FRET}}{\alpha_A^{FRET}(1-\beta E)+\beta E} \end{cases} \qquad \text{S23}$$

where $\beta = \mathcal{B}_D^{Dex} / \mathcal{B}_A^{Dex}$ is the ratio of Einstein absorption coefficients for donors and acceptors upon donor excitation.

Finally the molecular fraction of bound donors and acceptors can be calculated from Eq. S23 and the full information on binding stoichiometry is recovered:

$$\begin{cases} f_D^* = \dfrac{n_D^*}{n_D + n_D^*} = f_D^{FRET} \\ f_A^* = \dfrac{n_A^*}{n_A^* + n_A} = \dfrac{f_A^{FRET}}{1-f_A^{FRET}} \end{cases} \qquad \text{S24}$$

Therefore, the FRET efficiency E, bound donor fraction f_D^* and bound acceptor fraction f_A^* can be recovered with our method.

Description of the generalized method for multi-exponential donor and acceptor

In cases when fluorescence decays cannot be described by a mono-exponential model, the different components of the donor and acceptor fluorescence decays and their corresponding fractional contributions need to be established. This can be achieved from for instance, phasor plot analysis which resolves multiple harmonics (7, 8) of fluorescence measured from a donor-only sample (to resolve donor decay components) and from a measurement in the acceptor channel (to resolve acceptor decay components).

For a multi-exponential-decaying donor and acceptor pair, the time evolutions of the excited-state population of unbound donors and unbound acceptors can be described as follows:

$$\begin{cases} N_D(t) = \sum_x N_{Dx}(t) = N_D(0)\sum_x p_{Dx} e^{-(k_{rDx}+k_{nrDx})t} = N_D(0)\sum_x p_{Dx} e^{-\frac{t}{\tau_{Dx}}} \\ N_A(t) = \sum_y N_{Ay}(t) = N_A(0)\sum_y p_{Ay} e^{-(k_{rAy}+k_{nrAy})t} = N_A(0)\sum_y p_{Ay} e^{-\frac{t}{\tau_{Ay}}} \end{cases} \qquad \text{S25}$$

where x and y in the above equation represent the indices of the different decay components of donor and acceptor, respectively. p_{Dx} and p_{Ay} respectively represent the molecular fraction of donor from the component x and that of the acceptor from component y.

Here, we assume that each donor decay component interacts independently with each acceptor decay component with identical FRET efficiency E (9). Also, we assume that all donor components have the same spectral property. The same assumption for all acceptor components also applies here. The time evolution of the excited-state populations for bound donors and bound acceptors ($N_D^*(t)$ and $N_A^*(t)$) can then be written as:

$$\begin{cases} \dfrac{\mathrm{d}N_D^*(t)}{\mathrm{d}t} = \dfrac{\mathrm{d}\sum_x N_{Dx}^*(t)}{\mathrm{d}t} = -\sum_x \left(k_{rDx}+k_{nrDx}+k_{FRETx}\right) N_{Dx}^*(t) = -\sum_x \dfrac{1}{\tau_{Dx}^{FRET}} N_{Dx}^*(t) \\ \dfrac{\mathrm{d}N_A^*(t)}{\mathrm{d}t} = \dfrac{\mathrm{d}\sum_y N_{Ay}^*(t)}{\mathrm{d}t} = \sum_x k_{FRETx} N_{Dx}^*(t) - \sum_y \left(k_{rAy}+k_{nrAy}\right) N_{Ay}^*(t) = \sum_x \dfrac{E}{\tau_{Dx}^{FRET}} N_{Dx}^*(t) - \sum_y \dfrac{1}{\tau_{Ay}} N_{Ay}^*(t) \end{cases} \qquad \text{S26}$$

Furthermore we make use of the fact that:

$$p_{Dx}=\frac{N_{Dx}^{*}(0)}{N_{D}^{*}(0)}=\frac{N_{Dx}(0)}{N_{D}(0)}=\frac{n_{Dx}^{*}}{n_{D}^{*}}=\frac{n_{Dx}}{n_{D}} \qquad \text{S27}$$

Since the spectral properties of the different donor and acceptor states are constant, Eq. S13 still holds true. From Eqs. S13, S25, S26, S27 then, the time-evolution of the total excited-state population can be obtained for the donor and acceptor upon donor excitation:

$$\begin{cases} N_D^{tot}(t)=\overbrace{\mathcal{B}_D^{Dex}P^{Dex}n_D^{*}\sum_x p_{Dx}e^{-t/\tau_{Dx}^{FRET}}}^{N_D^{*}(t)}+\overbrace{\mathcal{B}_D^{Dex}P^{Dex}n_D\sum_x p_{Dx}e^{-t/\tau_{Dx}}}^{N_D(t)} \\ N_A^{tot}(t)=\overbrace{\mathcal{B}_D^{Dex}P^{Dex}n_D^{*}E\sum_{x,y}\frac{p_{Dx}p_{Ay}\tau_{Ay}}{\tau_{Ay}-\tau_{Dx}^{FRET}}(e^{-t/\tau_{Ay}}-e^{-t/\tau_{Dx}^{FRET}})+\mathcal{B}_A^{Dex}P^{Dex}n_A^{*}\sum_y p_{Ay}e^{-t/\tau_{Ay}}}^{N_A^{*}(t)} \\ \quad +\overbrace{\mathcal{B}_A^{Dex}P^{Dex}n_A\sum_y p_{Ay}e^{-t/\tau_{Ay}}}^{N_A(t)} \quad (\tau_{Ay}\neq\tau_{Dx}^{FRET}) \end{cases} \qquad \text{S28}$$

In what follows we will concentrate on the case for which $\tau_{Ay}\neq\tau_{Dx}^{FRET}$ for all *x* and *y* combination:

According to Eq. S28, and using the same calculation method as described from S16 to S21, we obtain:

$$\vec{\tau}_D=\frac{\sum_x p_{Dx}\tau_{Dx}\vec{\tau}_{Dx}}{\sum_x p_{Dx}\tau_{Dx}} \quad , \quad \vec{\tau}_D^{FRET}=\frac{\sum_x p_{Dx}\tau_{Dx}^{FRET}\vec{\tau}_{Dx}^{FRET}}{\sum_x p_{Dx}\tau_{Dx}^{FRET}} \qquad \text{S29}$$

$$\vec{\tau}_A=\frac{\sum_y p_{Ay}\tau_{Ay}\vec{\tau}_{Ay}}{\sum_y p_{Ay}\tau_{Ay}} \quad , \quad \vec{\tau}_A^{FRET}=\frac{\sum_{x,y} p_{Dx}p_{Ay}\tau_{Ay}(\tau_{Ay}\vec{\tau}_{Ay}-\tau_{Dx}^{FRET}\vec{\tau}_{Dx}^{FRET})/(\tau_{Ay}-\tau_{Dx}^{FRET})}{\sum_y p_{Ay}\tau_{Ay}} \qquad \text{S30}$$

It can be easily proven that Eq. S22 is still valid for the current case. Thus we obtain:

$$\begin{cases} f_D^{FRET}=\frac{n_D^{*}}{n_D+n_D^{*}}=\frac{\alpha_D^{FRET}\sum_x p_{Dx}\tau_D}{(1-\alpha_D^{FRET})\sum_x p_{Dx}\tau_{Dx}^{FRET}+\alpha_D^{FRET}\sum_x p_{Dx}\tau_D} \\ f_A^{FRET}=\frac{n_A^{*}}{n_A^{*}+(n_A^{*}+n_A)}=\frac{\alpha_A^{FRET}}{\alpha_A^{FRET}(1-\beta E)+\beta E} \end{cases} \qquad \text{S31}$$

On substitution into Eq S24, the true bound fractions of donor and acceptor can be obtained.

It should be noted that the method for obtaining active and passive donor phasors, as well as the FRET efficiency *E*, for multi-exponential fluorophores is similar, but not identical, to the mono-exponential case. We have assumed in the current derivation that *E* is the same for all possible combinations of donor and acceptor components, and therefore, according to Eq. S12, for any donor component *x*, the following equation holds:

$$\tau_{Dx}^{FRET}=\tau_{Dx}(1-E) \qquad \text{S32}$$

This leads to a donor phasor trajectory for different *E* values, which, if multi-exponential decays prevail, lies on the inside of the universal circle rather than on the circle itself. In principle, the same construction methods as explained for the single exponential decay can then be used to recover the active and passive donor phasors.

3) Dissociation constant

The dissociation constant of the interacting FRET pair is defined as:

$$K_d=\frac{([\mathrm{A}]-[\mathrm{AD}])([\mathrm{D}]-[\mathrm{AD}])}{[\mathrm{AD}]} \qquad \text{S33}$$

where [A] and [D] are respectively the total acceptor and total donor concentration. [AD] is the concentration of the bound donor – acceptor complex. ([A]-[AD]) and ([D]-[AD]) are respectively the concentration of unbound

acceptor and unbound donor. Equation S33 assumes a 1:1 interaction stoichiometry between donor and acceptor. The total concentration of donor and acceptor can be written as follows:

$$\begin{cases} [\mathrm{A}] = f_D^* \cdot [\mathrm{D}] / f_A^* \\ [\mathrm{D}] = f_A^* \cdot [\mathrm{A}] / f_D^* \end{cases} \qquad \text{S34}$$

Therefore Eq. S33 can be expressed as:

$$K_d = [\mathrm{D}]\frac{(1-f_A^*)(1-f_D^*)}{f_A^*} = [\mathrm{A}]\frac{(1-f_A^*)(1-f_D^*)}{f_D^*} \qquad \text{S35}$$

According to Eq. S34 and S35, if one value among K_d, [D] and [A] is known, the other two parameters can be recovered.

In cases when there are unlabeled samples (molecules not labelled with fluorophores) in the system, the unlabeled molecules may bind the labelled binding partner with the same affinity as the labelled ones. Therefore the real K_d can be expressed as:

$$K_d = \frac{([\mathrm{A}]+[\mathrm{A'}]-[\mathrm{AD}]-[\mathrm{A'D}]-[\mathrm{AD'}]-[\mathrm{A'D'}])([\mathrm{D}]+[\mathrm{D'}]-[\mathrm{AD}]-[\mathrm{A'D}]-[\mathrm{AD'}]-[\mathrm{A'D'}])}{[\mathrm{AD}]+[\mathrm{A'D}]+[\mathrm{AD'}]+[\mathrm{A'D'}]} \qquad \text{S36}$$

where A' and D' denote the proteins, which were not successfully labelled with acceptor and donor fluorophores, respectively. The apparent K_d in this case is calculated with Eq. S35, and is obviously larger than in the absence of competitors.

In the specific case where only one type of unlabeled sample exists in the system, for instance D', then the real K_d can be expressed as

$$\begin{aligned} K_d &= \frac{([\mathrm{A}]-[\mathrm{AD}]-[\mathrm{AD'}])([\mathrm{D}]+[\mathrm{D'}]-[\mathrm{AD}]-[\mathrm{AD'}])}{[\mathrm{AD}]+[\mathrm{AD'}]} \\ &= \frac{((1-f_A^*)\cdot[\mathrm{A}]-[\mathrm{D'}]\cdot f_D^*)(1-f_D^*)}{f_D^*} \end{aligned} \qquad \text{S37}$$

It can be seen from Eq. S37 and S34, if two parameters among [A], [D] and K_d are known, the third and the concentration of the competitor can all be recovered.

It is often possible to choose an acceptor channel (acceptor excitation / acceptor emission) where negligible cross-excitation of donors occurs, *e.g.* by using an excitation wavelength that is sufficiently red-shifted. The fluorescence intensity image measured in the acceptor channel provides a measure of the concentration variation of the acceptor. By carrying out an intensity calibration using a group of standard acceptor sample with known concentration, the acceptor concentration [A] can be recovered from an experimental sample *via* an intensity measurement in the acceptor channel.

In cases where such a calibration is not possible, the acceptor intensity channel still offers information on *relative* concentrations, *i.e.* $\gamma[\mathrm{A}]$ where γ is an unknown proportionality constant. According to Eq. S35, one can then recover γK_d. On the other hand, if γK_d is known and if a single competitor is present in the system, then *via* Eq. S37 γ[competitor] can be obtained.

S2. Sample preparation

1) Oregon green – glutathione (OG-GSH) and mCherry-Glutathione S-transferase (mCherry-GST) preparation

A bacterial expression plasmid encoding a 6X His-tagged fusion of mCherry and S. japonicum glutathione S-transferase (GST), separated by a Tobacco etch virus (TEV) protease cleavage site was constructed by PCR amplification of the coding sequences and their ligation into the pQE10 plasmid (Qiagen). The sequence of the fusion protein encoded by this mCherry_TEV_GST_pQE10 plasmid (Ron Lab Stock number UK910) is provided below (mCherry in red, GST in green):

MRGSHHHHHHTDLVSKGEEDNMAIIKEFMRFKVHMEGSVNGHEFEIEGEGEGRPYEGTQTAKLKVTKGG
PLPFAWDILSPQFMYGSKAYVKHPADIPDYLKLSFPEGFKWERVMNFEDGGVVTVTQDSSLQDGEFIYKV
KLRGTNFPSDGPVMQKKTMGWEASSERMYPEDGALKGEIKQRLKLKDGGHYDAEVKTTYKAKKPVQL
PGAYNVNIKLDITSHNEDYTIVEQYERAEGRHSTGGMDELYKENLYFQGSMSPILGYWKIKGLVQPTRLLL
EYLEEKYEEHLYERDEGDKWRNKKFELGLEFPNLPYYIDGDVKLTQSMAIIRYIADKHNMLGGCPKERAE

ISMLEGAVLDIRYGVSRIAYSKDFETLKVDFLSKLPEMLKMFEDRLCHKTYLNGDHVTHPDFMLYDALDV VLYMDPMCLDAFPKLVCFKKRIEAIPQIDKYLKSSKYIAWPLQGWQATFGGGDHPPKSDLVPRGSPGISGG GGGILDSMGRLELKLN

A mammalian expression version of this chimera (mCherry_TEV_GST_pCDNA3, Ron Lab Stock number 1279) was constructed by cloning the coding sequence into pCDNA3 vector (Invitrogen).

The mCherry_TEV_GST_pQE10 plasmid was transformed into E. coli M15 (pREP4) strain, induced with 1 mM IPTG and purified by sequential Ni chelate chromatography followed by size exclusion chromatography (S200, GE Healthcare).

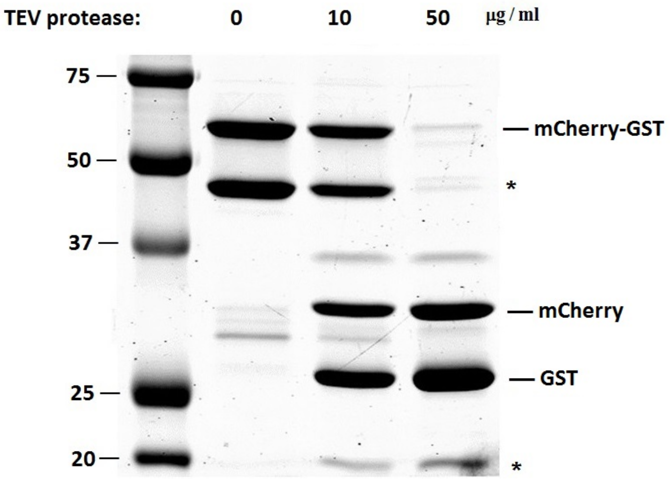

Figure S2. Coomassie gel of the H6-mCherry-GST fusion protein purified from E. coli by Nickel chelate chromatography. Where indicated, the protein had been incubated with the TEV protease to separate the mCherry from the GST moiety. Like other dsRED fluorescent proteins, when heated, mCherry undergoes spontaneous cleavage of the peptide backbone (due to hydrolysis of the acylimine C=N bond in the chromophore, (10, 11)), this accounts for emergence of N-terminally-deleted species of mCherry during sample preparation for the SDS page (marked with asterisks). Such species are not present in the protein preparations used in the FRET measurements

To produce glutathione bound to Oregon Green (OG-GSH), reduced glutathione (5mM, G6529, Sigma, Dorset, UK) was incubated with Oregon green 488 iodoacetemide (2.5mM, O-6010, Life-technologies, Paisley, UK) in potassium phosphate buffer (50mM, pH 7.5) for 2 hours, followed by quenching the reaction by 2-mercaptoethanol (25mM). The sample was then acidified with TFA and bound to a C18 sep-pak column washed in 0.1% TFA and eluted in 30% acetonitrile 0.1% TFA. Cross-linked OG-GST was then purified by analytical reverse phase HPLC chromatography using a Poroshell 120 C18 column (Agilent Technologies). The purified OG-GSH solution was lyophilized into powder to enable the preparation of OG-GSH solution in different solvents.

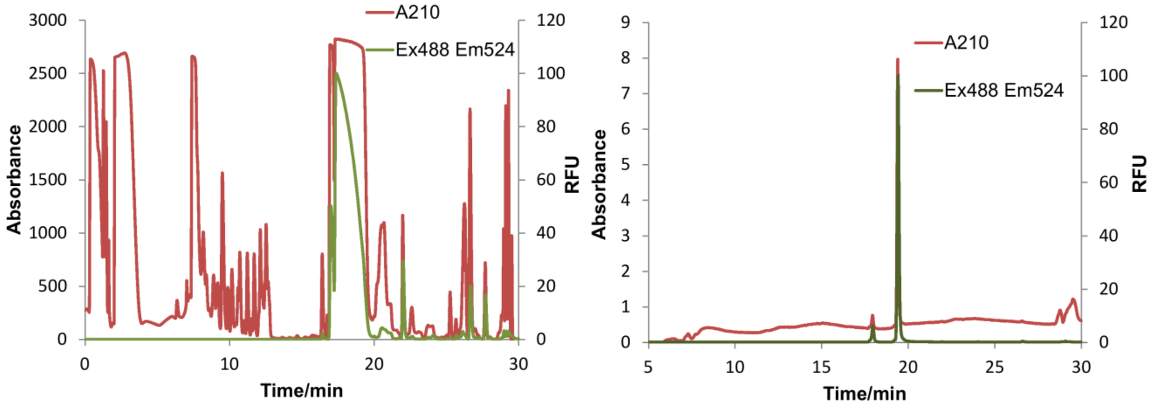

Figure S3. HPLC purification of OG-GSH. A. Chromatogram of reverse phase C18 column HPLC purification of 1.81 μM OG-GSH from acidified reaction mixes. The peak between 17 and 19 minutes of retention time was collected and dried. B. Chromatogram of 0.9 nM purified OG-GSH injected on reverse phase C18 HPLC to check purity. Absorbance at 210 nm is plotted on the left and relative fluorescence excited at 488 nm and emitted at 524 nm is plotted on the right.

According to the phasor plot for OG-GSH and mCherry-GST solution mixtures in microfluidic device (Figure S14a in Section S5 – also see Section S2, 4) for details), it is appropriate to describe the fluorescence

decay of OG-GSH and mCherry-GST with single-exponential model. This is because the end of the donor channel phasor cloud at the high-lifetime side centers near the universal circle, and the FRET passive acceptor phasor also lies near the universal circle; these show that the fluorescence decays of OG-GSH and mCherry-GST are approximately single-exponential.

2) Cell Culture, transfection and sample preparation:

HEK293T cells were cultured in DMEM supplemented with 10% FBS (referred to as 'HEK medium' in the following) and maintained at 37 °C with 5% CO_2. Cells were transfected in the culture flask with the mCherry_TEV_GST_pCNDA3 plasmid (5 g of DNA/1 X 106 cells) using the Neon transfection system (Life-technologies) following the manufacturer's protocol, and analyzed 24-48 hours post-transfection.

24-48 hours after transfection, HEK293T cells were seeded in 8 well Lab-Tek chambered coverslips (Thermo Scientific). The cells were then placed in culture again with HEK medium for 24 hours. For permeabilization, 60 µg/ml saponin (Sigma-Aldrich) (water solution, stock concentration 2 mg/ml) was added into the medium and mixed with pipette for 5 mins (12). OG-GSH solution (prepared in PBS, pH adjusted to 7-7.5, stock solution concentration 325 µM) was added to the medium after the addition of saponin, therefore the permeabilization status of the cells could be monitored by measuring the fluorescence in the donor channel. Due to the cell membrane permeabilization, the endogenous GSH, which are small molecules, diffuse into the medium and are depleted from within cells.

The solution of reduced glutathione (GSH) was prepared 48 hours prior to imaging by dissolving GSH powder into Milli-Q water. The pH of the GSH solution was then adjusted to 7-7.5, using NaOH. The stock GSH solution concentration is 10 mM.

3) Microfluidic chip fabrication

Microfluidic devices were fabricated by soft lithography (13, 14). Briefly, high-resolution photomasks were designed with CAD software (Autodesk) and printed on a film (MicroLithography Services). Subsequent steps were performed in a cleanroom (class 100). Three inch silicon wafers (100) (Si-Mat) were spin-coated with SU-8 2100 (Microchem Corp). This step resulted in a resist thickness of 150 µm. Spin-coated wafers were then soft-baked and exposed to UV-light using a MJB4 mask aligner (Suss MicroTec). After post-exposure baking, wafers were developed in a solution of propylene glycol monomethyl ether acetate (Sigma-Aldrich). Developed wafers (master molds) were washed with isopropanol and de-ionized water. Finally, the wafers were hard-baked at 150 °C for 5 minutes.

Master molds were coated with trichloro(1H,1H,2H,2H-perfluorooctyl)silane (Sigma-Aldrich) by chemical vapour deposition. Silanized master molds were used for the cast molding of PDMS elastomer as described by Xia *et al.*(13) Sylgard 184 (Dow Corning) was used for the formation of PDMS molds. PDMS precursor and curing agent were mixed in a mass ratio of 10 to 1. The mixture was cast onto the treated master mold placed in a Petri dish, degassed and cured in an oven for at least 2 hours at 65 °C. A thin PDMS layer was produced using the same process and used for bonding in an oxygen plasma cleaner (Diener Electronics) with the fluidic structures. Polytetrafluoroethylene tubing (PTFE; I.D. 200 µm, O.D. 400 µm) was inserted in a custom-made side channel and sealed using PDMS. The serpentine channel width was 150 µm and each lane was 3 mm long.

4) Production of droplets with varying concentrations of donor and acceptor

The solutions of mCherry-GST and OG-GSH used for the microfluidic experiment were prepared in HEPES buffer (50 mM HEPES and 150 mM NaCl, pH 7.2). The concentration of mCherry-GST solution was 68.4 µM, and the concentration of OG-GSH solution was 25.1 µM.

Droplets with varying ratios of donor/acceptor were produced by sampling from an open well source (part of a 384 well-plate) using a negative pressure technique with aligned capillaries (15). The overall setup is shown in Fig. S4.

The well contained initially 20 µL mCherry-GST together with a magnetic stir bar (Fisher Scientific). Droplets were formed by placing a PTFE tubing (I.D. 100 µm, O.D. 400 µm) into a pipette tip of matching dimensions (StarLab, 200 µL round, bottom I.D. 360 µm, top I.D. 5 mm). 20 µL of an oil phase (HFE-7500 + 1 wt% EA surfactant) was dispensed into the tip. Applying negative pressure to the PTFE tubing using a syringe pump (Chemyx, Fusion 200) in withdrawal mode resulted in the simultaneous withdrawal of continuous and dispersed phase to produce monodisperse droplets with a volume of 3 nL (at withdrawal flow rate of 1 µL/min). A gas-tight syringe (SGE) containing OG-GSH was used to inject the donor dye to the well and the stir bar ensured complete mixing. After an initial production of droplets containing only the acceptor solution for 10 seconds, the withdrawal flow rate for droplet generation was decreased to 0.8 µL/min and the injection of donor solution initiated. The flow

rate for injection was 50 µL/min for 80 seconds. A third tubing connected to a gas-tight syringe (SGE) was used to withdraw solution from the well using a syringe pump operating in withdrawal mode (at 50 µL/min) in the same time as the donor injection. This configuration ensured a swift switch between acceptor and donor solutions.

After the droplets with varying stoichiometry were produced, the tubing was removed from the tip and inserted into a side port of the microfluidic chip before being sealed with degassed PDMS and cured on a hot plate at 90℃ for 5 minutes. Droplets were subsequently transferred to the chip by pushing them back (at 1 µL/min) and stopping them in the serpentine channel using the bright-field view of a microscope (Brunel Microscopes) as a feedback. Both inlet and outlet tubing were then clamped and the chip immersed in a de-ionized water bath to minimize evaporation and motion of the droplets within the channels. For imaging, the chip was taken out of the water bath, dried and placed onto the microscope stage. During the measurement time (approximately 30 minutes), droplets were immobile.

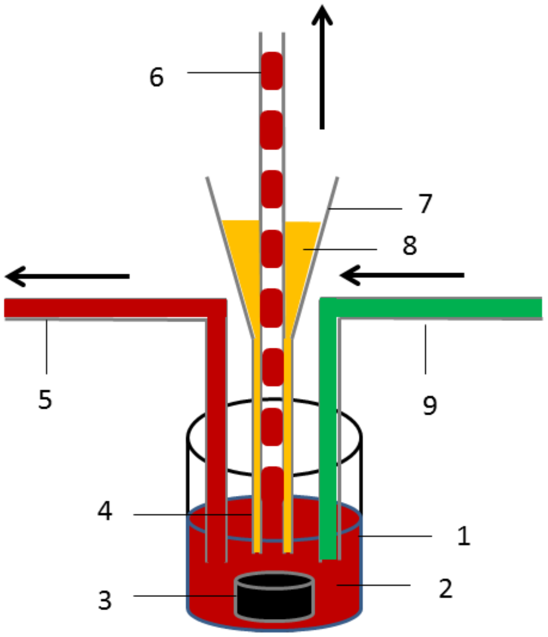

Figure S4. Schematic of the setup for producing droplets containing varying concentrations of donor/acceptor mixtures. 1. Well of a 384 well-plate. 2. mCherry-GST solution. 3. Magnetic stir bar. 4. PTFE tubing in which droplets are stored. 5. Tubing used for withdrawing solution from the well. 6. 3 nL droplets with increasing concentration of donor dye. 7. Pipette tip. 8. HFE-7500 with 1 wt% EA surfactant. 9. Tubing used to inject Oregon Green-GSH solution into the well. Arrows indicate the direction of flows in the respective tubings.

S3. Experimental methods

1) Data acquisition with FLIM-TCSPC system

The FLIM measurements were carried out on a custom-made FLIM-TCSPC system (16), using a super-continuum laser (SC450, Fianium) with a pulse repetition rate of 20MHz, a confocal scanning unit (FluoView 300, Olympus) coupled with an inverted microscope frame (IX70, Olympus), and a time-correlated single photon counting (TCSPC) system (Becker & Hickl GmbH). The detection window of the TCSPC system was set to 25 ns, separated into 256 time bins. Each FLIM image has 256×256 pixels. The excitation wavelength was selected by using an acousto-optic tunable filter (AOTFnC-400.650, Quanta Tech) and an excitation filters (to improve the wavelength selection), and the fluorescence from the sample was imaged through appropriate emission filters. The donor excitation wavelength was chosen by selecting the AOTF wavelength as 472 nm, and applying a band-pass filter (BP470/490, Chroma Technology). The acceptor excitation wavelength was chosen by selecting the AOTF wavelength as 575 nm, and applying a band-pass filter (BrightLine FF01-585/29, Semrock). The donor fluorescence was detected with a band-pass filter centered at 520 nm (BrightLine FF01-520/35, Semrock). The acceptor fluorescence was detected using a long pass filter cutting at 630nm (RG-630 Long Pass Filter, Edmund Optics).

For our experiments, the data acquisition time was 200s for each FLIM image (10 cycles, 20s per cycle). The photon detection rate was kept below 5% of the laser repetition rate in order to avoid photon pile-up (17).

2) Data processing and discussions

All data processing was performed using a custom-written MATLAB code (available from our website laser.ceb.cam.ac.uk). Because the TCSPC detection window was set to 25 ns, the phasors were calculated from the Fourier transform at 40 MHz as previously presented (18).

The IRF calibrations were performed *via* measurements of decays from standard samples (2), using Rhodamine 6G (R6G) and Alexa 647. The fluorescence decay from R6G was measured in both donor and FRET channels, and the fluorescence decay from Alexa 647 was measured in the acceptor channel. The lifetimes of the standard samples are obtained by fitting to the decay curve. These measurements permitted calibration of respective phasors for all three channels.

In Fig. 1c-1f in the main article, the time-resolved data were spatially binned (using a 5×5 pixels kernel convolution) in order to increase photon counts in each binned pixel to a final average photon count around 14,000. In this case of low photon count, no background compensation was performed (see simulations in Section S4 for details and reasoning on this procedure: In short, the background subtraction procedure introduces bias and noise at low count levels, whereas benefits are derived, when signal levels are large).

In Fig. 1g and 1h, spatial binning using convolution with a 13×13 pixels kernel was applied (corresponding to a micro-channel width of around 13 pixels in measured images). Here, the photon counts obtained in each binned pixel was averagely around 90,000, and a background correction was performed. Background levels were estimated by averaging the photon counts in the first 8 time bins and subtracted from the corresponding fluorescence decay curve, prior to calculations of phasors.

In Fig. 2, images were spatially binned using a 5×5 pixels kernel convolution. The average photon counts in the binned pixel were below 20,000; no background correction was applied. The influence of autofluorescence on the FLIM data and the prevention of autofluorescence related artifacts have been discussed previously (1, 19, 20). We estimated the autofluorescence levels from non-transfected cells (treated identically to those that were transfected) and compensated for this by exploiting the vector addition properties of phasors (19). The cells were measured in the donor, FRET and acceptor channels respectively, with identical acquisition parameters as used for the transfected cells. The phasors representing the autofluorescence signal in the three channels were obtained as previously presented (19, 1), and average photon counts from the autofluorescence determined. We assumed that the autofluorescence always contributed the same decay component to the measured signal in each pixel (same amount of photons and same phasors) and could thus be removed by phasor subtraction.

For our experiments, β was obtained with calibration samples. Here, a set of samples containing known concentrations of OG-GSH and mCherry-GST was prepared (in 50 mM HEPES and 150 mM NaCl, pH 7.2, 37℃) and the bound fractions of donors and acceptors were measured using our method. Figure S5 shows phasor plots for these samples.

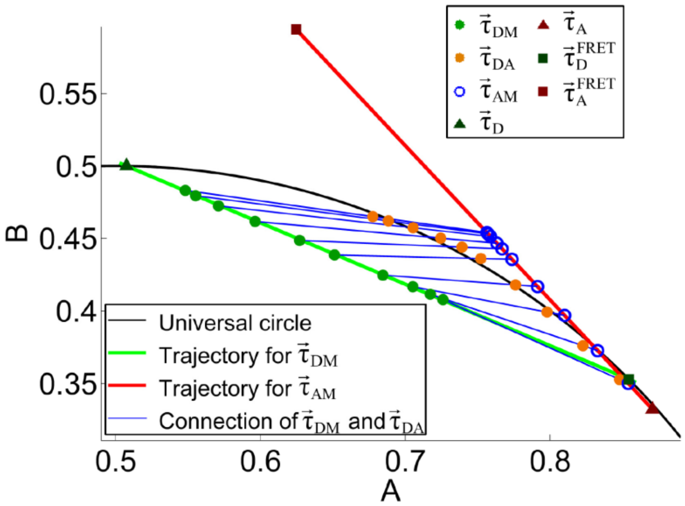

Figure S5. Phasor plot of standard sample fluorescence for the measurement of β.

With Eq. S23 and Eq. S34, a value for β could be determined for each sample point. A value of 5.03 was obtained for β by averaging all β values obtained from all data points. This is possible when calibration data is available. If this is not the case, β can also be obtained from dye absorption spectra. It should be noted that measurement of the β value from dye solutions should be performed under similar environmental conditions as those prevailing in cells (pH, temperature, salts *etc.*) to be representative (5).

In Fig. 2c, 2f and 2g, the acceptor concentrations were estimated from the acceptor intensity measurements performed in the acceptor channel upon direct acceptor excitation. Here, for intensity/concentration calibration, the intensity of different mCherry-GST solutions with different known concentrations were measured, with identical acquisition parameters as when performing the cell experiments. Photon counts for mCherry-GST were measured as a function of concentration and found to obey a linear relationship (as shown in Fig. S6, note log scale for x-axis. Data: black squares, fit: red line).

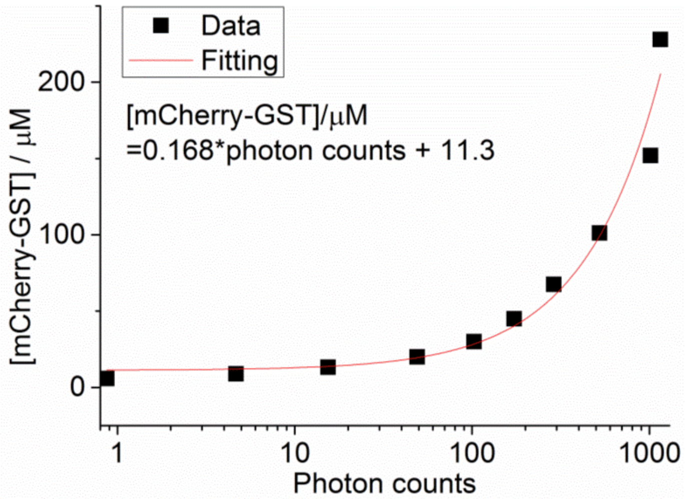

Figure S6. Acceptor intensity calibration for the recovery of acceptor concentration in cells.

The fitted parameters were subsequently used to determine the concentration of mCherry-GST from the photon count measured in the acceptor channel.

Knowing the phasor of the FRET passive donors, the FRET efficiency can be recovered in every pixel (20). Figure S7 shows corresponding FRET images for both microfluidic and cell data. Clearly the FRET efficiency is homogeneous across the sample, which is in good agreement with the phasor plots shown in Section S5 (donor channel phasors approximately lie on the same line, indicating that the FRET efficiency in the sample is constant). Figure S7a shows that when donor bound fraction is low (lower microfluid channels shown in the image), the recovered pixel-based FRET efficiency has low accuracy and precision because of the proximity of the active and passive donor channel phasors. On the assumption of a constant *E* across the image, the full strength of the phasor plot method comes into play as then information from all pixels can be used simultaneously and FRET efficiency recovered globally. This leads to the excellent robustness and accuracy of our method for a large dynamic range.

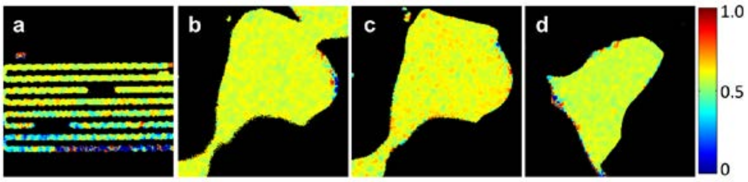

Figure S7. Recovered FRET efficiency E in different pixels. a: FRET efficiency image of the microfluidic experiment (corresponding to Fig. 1c-1f in main article). b: FRET efficiency image for cell data (corresponding to Fig. 2a-2c in main article). c: FRET efficiency image for cell with extra GSH (corresponding to Fig. 2d-2g). FRET efficiency image for cell data (corresponding to Fig. 2h-2k in main article).

S4. Simulations

Using MATLAB simulations, we generated TCSPC images with 256×256 pixels made of 256 individual blocks of 16×16 pixels. Within a single block, the signal from a specified concentration of donor (OG-GSH) and acceptor (mCherry-GST) was simulated and the noise in each pixel generated independently. Figure S8 shows the simulated donor and acceptor concentration in each block and the corresponding expected bound fractions (shown as a false color scale), using K_d=27 µM.

In each pixel, the fluorescence decay was obtained first, by computing the exponential decays using 4.00 ns for OG and 1.57 ns for mCherry, and a FRET efficiency of 60.0% (similar to the parameters obtained experimentally). Second, the decay was convolved with a simulated Gaussian instrumental response function (IRF) with a shift of 1.55 ns and a FWHM of 0.35 ns, similar to that obtained experimentally. Using a laser repetition rate of 20 MHz,

the decay curve was cropped after 50 ns position and the part of the decay after 50 ns was added to the cropped decay curve to mimic the influence of the incomplete decay fluorescence from the previous pulse.

The remaining decay was cropped between 0-25 ns and binned into 256 time bins. A constant background noise with amplitude of 0.1% of the peak intensity was then added to the decay curve, simulating the effect of background from the after-pulsing and stray light, as typically obtained experimentally. The curve was then normalized and multiplied by the specified total photon counts. After that, Poisson noise was added to the decay curve. Finally, the photon counts in each time bin were rounded to integers, and bins with negative photon counts were assigned a value of 0.

The simulated FLIM data for both the reference sample and the FRET sample were generated (using a lifetime of 3.85 ns for the reference sample, similar to that of R6G). The FLIM data of the FRET sample were generated for the three different detection channels, *i.e.* donor channel, FRET channel, and acceptor channel. For the FRET channel signal, the amount of donor bleed-through used was preliminarily estimated according to the spectrum and quantum yield of mCherry and OG fluorophore. The simulated FLIM data were then analyzed with our method.

We furthermore performed simulations for FRET pairs with lower FRET efficiency and a smaller separation between passive donor and passive acceptor lifetimes. We chose eGFP and mCherry, respectively, as a representative donor and acceptor pair. The passive donor lifetime was chosen as 2.56 ns (21), and active donor lifetime 2.00 ns (FRET efficiency 21.9%). The passive mCherry lifetime was still chosen as 1.57 ns. β=8 was used for the simulation (according to spectra and quantum yield values from Semrock SearchLight). The lifetime of the reference sample was chosen as 2.3 ns. Dissociation constant and donor / acceptor concentrations in each of the image blocks were set equal to those used for OG / mCherry simulations.

Figure S9 shows the recovered images for donor and acceptor bound fraction, and dissociation constants using our method.

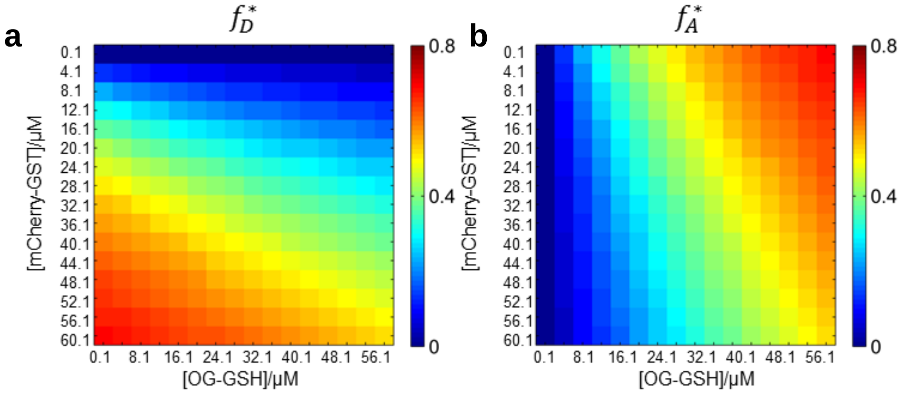

Figure S8. Expected bound fraction of donor and acceptor for the simulated sample.

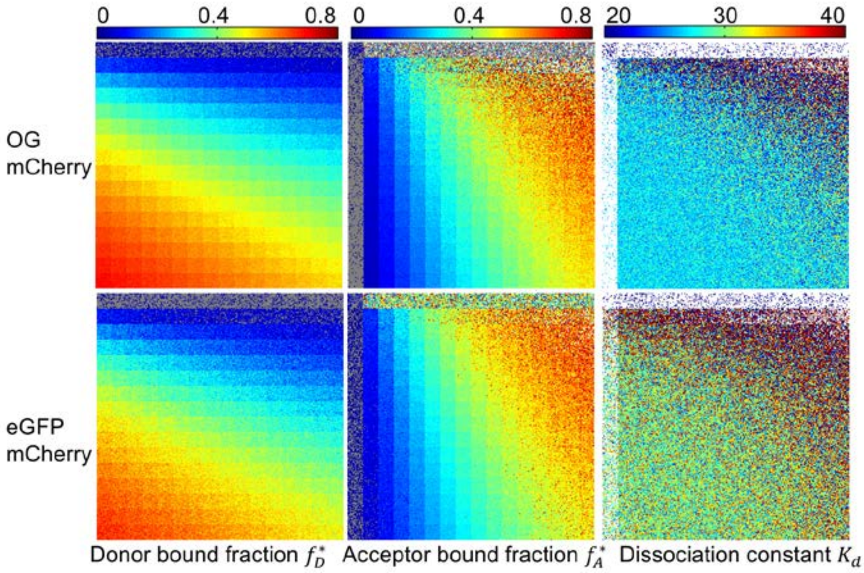

Figure S9. Recovered bound fractions of donor and acceptor, and recovered dissociation constants, for both the OG-mCherry pair and the eGFP-mCherry pair. The recovered FRET efficiency is E=(60.0±0.3)% for OG-mCherry

pair, and E=(22.0±0.3)% for eGFP-mCherry pair. For the f_D^* and f_A^* images, recovered bound fraction below 0 are shown as grey, bound fractions above 1 as white. For the K_d image, the white color indicates that f_D^* and f_A^* recovered for the specific pixel were not both within the range of 0-1. Decays used for the simulation were generated with 20,000 total photon counts, which is comparable to what is obtained in experiments in 5×5 binning mode.

In Fig. S9, background corrections were performed for the analysis of the OG / mCherry pair, but not for the eGFP / mCherry pair. Reasons will be discussed in following sections. The recovered f_D^*, f_A^* shown in Fig. S9 are in good agreement with the expected values shown in Fig. S8, apart from when either donor concentration or acceptor concentration are too small. The recovered K_d values also agree with the expected value (K_d=27 μM) in most cases. Next, we present an analysis of errors of the recovered parameters .

We investigated the effect of the total photon counts and the background correction on the recovered values of f_D^*, f_A^* and K_d. Mean values and standard deviations of the recovered parameters were obtained and compared with their expected values (see Fig. S10-S12), corresponding to the concentration maps shown in Figure S8.

The background correction was performed as previously described (see section S3,2). Figure S10-S12 shows that, when the number of photon counts is large, background correction results in better accuracy of recovered f_D^*, f_A^* and K_d; whereas when photon counts are low, the background correction will adversely affect the results and introduce bias and noise.

For the recovered f_D^* (Fig. S10), when acceptor concentration is very low (corresponding to the first row of the image), the accuracy of the recovered mean f_D^* for each block is poor. This is in agreement with the idea that the donor channel phasors ($\vec{\tau}_{DM}$) lies very close to that of the passive donor phasor ($\vec{\tau}_D$). Therefore, the determination of f_D^* value becomes very inaccurate, with many of them below 0. Similarly, the recovered f_A^* is not accurate when donor concentration is very low (see the first column of Fig. S11).

The accuracy and precision of the recovered f_A^* are relatively lower at the region where [D]/[A] is high. In this case, the contribution from the donor bleed-through in the FRET channel is dominant compared to the contribution from acceptor fluorescence signal. Therefore the FRET channel phasor $\vec{\tau}_{DA}$ lies close to the donor channel phasor $\vec{\tau}_{DM}$, which geometrically reduces the accuracy and precision with which $\vec{\tau}_{AM}$ can be recovered. Since the dissociation constant is calculated from f_D^* and f_A^*, the accuracy and precision of K_d are also lower when [D]/[A] is high. This is in agreement with the experimental results shown in Fig. 1 in the main article.

Comparing the simulations for the OG / mCherry and eGFP / mCherry pairs, one sees that for former, at the same photon count, a higher accuracy and precision is obtained for the recovered f_D^*, f_A^* and K_d. For eGFP / mCherry, it is evident that a higher photon count is required before background correction improves the accuracy of recovered parameters.

In conclusion, the simulated data for OG / mCherry pair show that the MC-FLIM-FRET method can recover f_D^*, f_A^* and K_d with good fidelity for a large dynamic range of [D] and [A], with the photon counts that are typically available in our experiment. Our simulation for eGFP and mCherry shows that the method is reliable also for pairs featuring a lower FRET efficiency and a less pronounced separation between the donor and acceptor lifetimes. In practice, obviously, we recommend the use of fluorophore pairs with high FRET efficiency for the quantitative recovery of stoichiometric information. Also, we note that it is useful to perform background correction when the photon counts are high, whereas the background correction should be avoided when photon counts are low because it introduces bias. For our OG-GSH and mCherry-GST experiments, the background corrections were performed when photon counts for each binned pixel were higher than 20,000, otherwise no background correction was performed. For the eGFP and mCherry pair, background corrections are not recommended when photon counts in each binned pixel are less than 80,000.

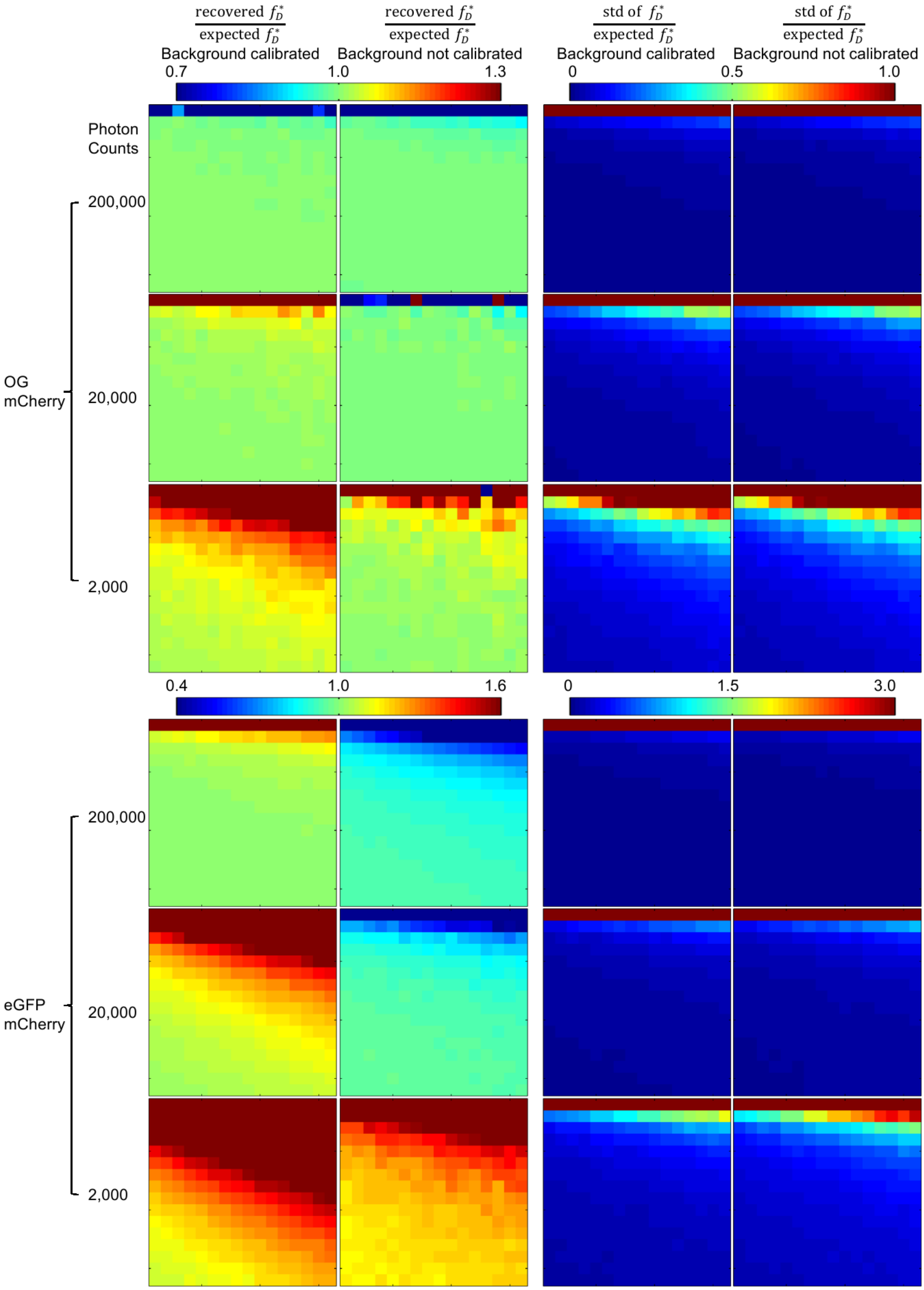

Figure S10. Mean value and standard deviation of the recovered donor bound fraction (f_D^*) in each simulated block, obtained from datasets with different photon counts for both the OG / mCherry and eGFP / mCherry pair. The total photon count per pixel is shown above each column. The effect of the background correction is also shown here.

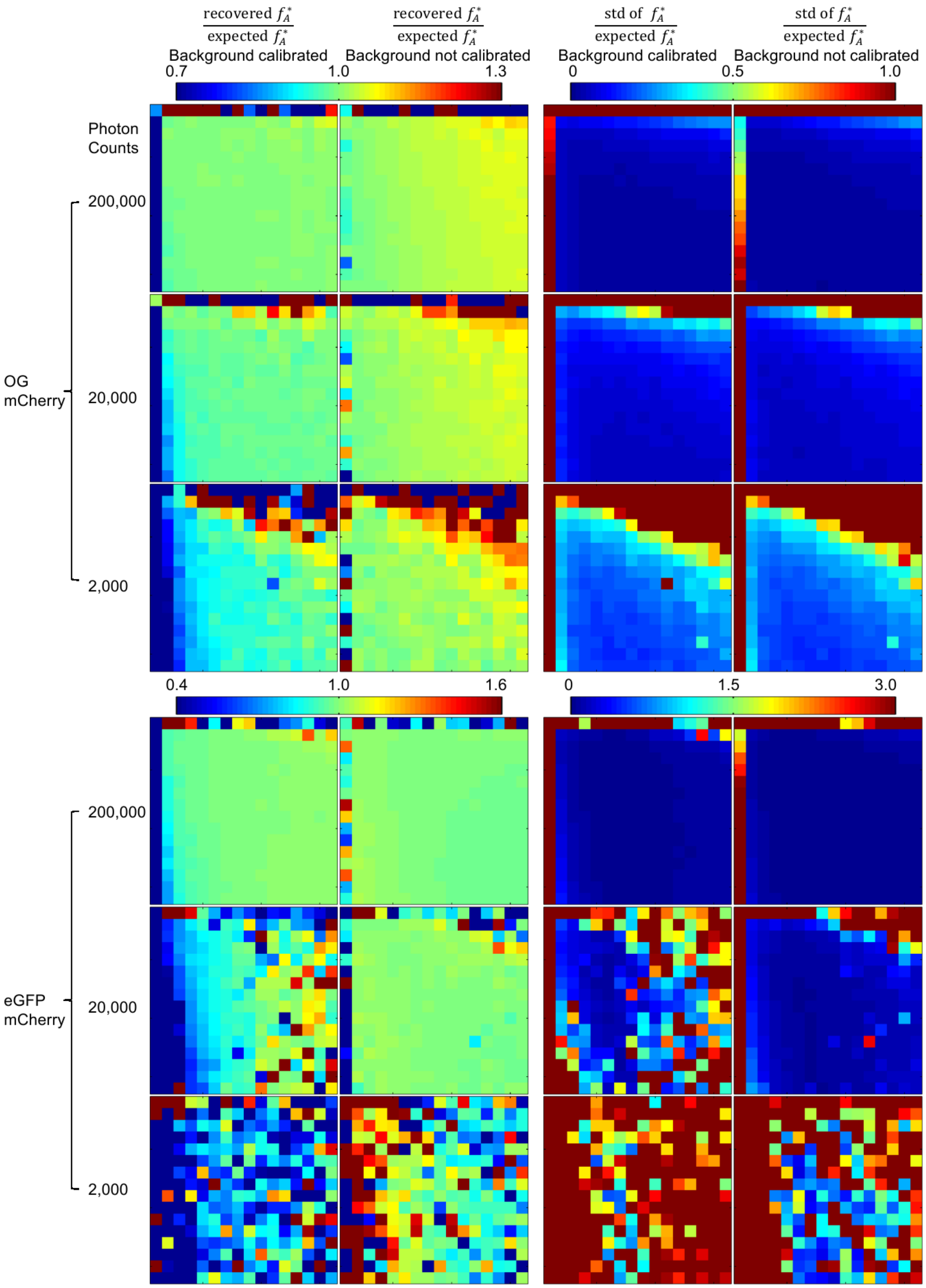

Figure S11. Mean value and standard deviation of the recovered acceptor bound fraction (f_A^*) in each simulated block, obtained from datasets with different photon counts for both the OG / mCherry and eGFP / mCherry pair. The total photon count per pixel is shown above each column. The effect of the background correction is also shown here.

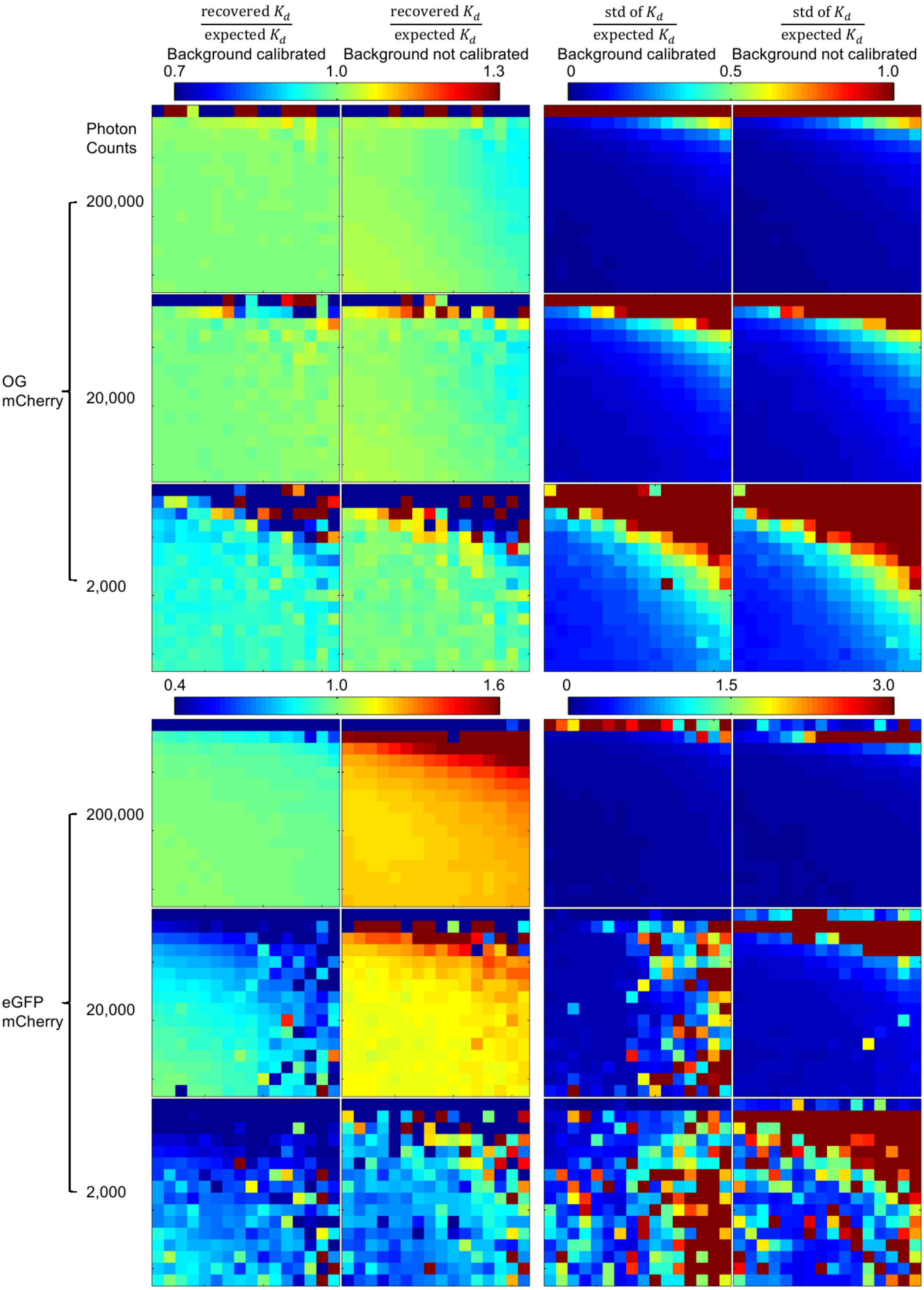

Figure S12. **Mean value and standard deviation of the recovered dissociation constant (K_d) in each simulated block, obtained from datasets with different photon counts for both the OG / mCherry and eGFP / mCherry pair.The total photon count per pixel is shown above each column. The effect of the background correction is also shown here.**

S5. Supporting figures for phasor plots

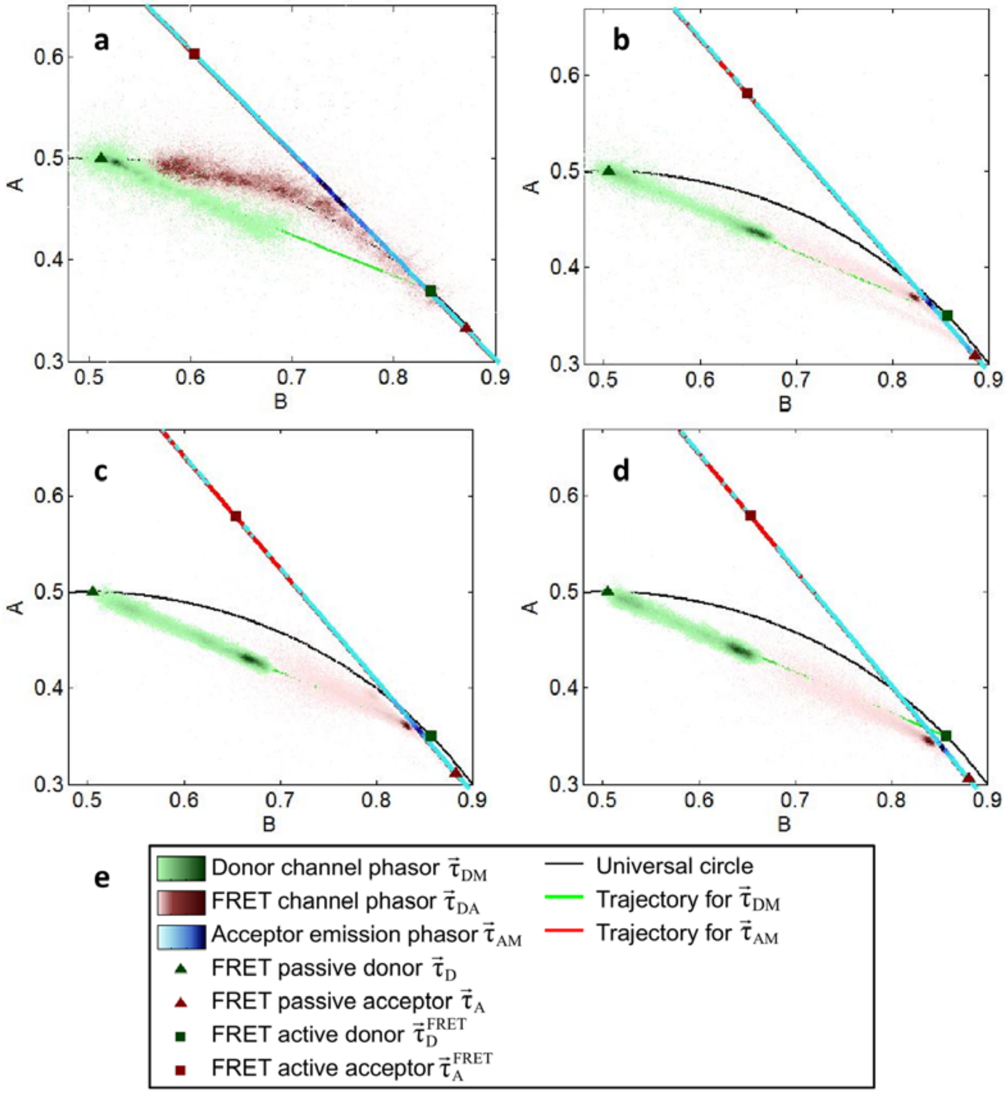

Figure S13. Phasor plots obtained from the microdroplet and cell analysis. a: Phasor plot for microfluidic data (corresponding to Fig. 1c-1f in main article). b: Phasor plot for cell data (corresponding to Fig. 2h-2k in main article). c: phasor plot for cell data (corresponding to Fig. 2a-2c in main article). d: phasor plot for cell with extra GSH (corresponding to Fig. 2d-2g). e: legend for phasor plots. For $\vec{\tau}_{DM}$, $\vec{\tau}_{DA}$ and $\vec{\tau}_{AM}$, darker color indicates higher density of phasors plotted in the corresponding region.

S6. Nomenclature

Table S1. Terminology used in the paper

donor channel	excited at the donor wavelength and detected by donor emission channel
FRET channel	excited at the donor wavelength and detected by acceptor emission channel
bound	donor/acceptor binding to acceptor/donor
unbound	donor/acceptor not binding to acceptor/donor
active	donor/acceptor undergoing FRET
passive	donor/acceptor not undergoing FRET
[A]	total acceptor concentration (including bound and unbound acceptors)
[D]	total donor concentration (including bound and unbound donors)
[AD]	concentration of the bound donor – acceptor complex
A	phasor coordinate representing the imaginary part of the Fourier transform

B	phasor coordinate representing the real part of the Fourier transform
E	FRET efficiency (ranging from 0 to 100%)
f_A^{FRET}	molecular fraction of active acceptors
f_D^{FRET}	molecular fraction of active donors
f_A^*	molecular fraction of bound acceptors
f_D^*	molecular fraction of bound donors
$I_A^{tot}(t)$	fluorescence intensity (number of photons emitted) from the acceptor
$I_D^{tot}(t)$	fluorescence intensity (number of photons emitted) from the donor
$I^{AexAem}(t)$	fluorescence intensity detected in the acceptor channel upon excitation at the acceptor wavelength
$I^{DexAem}(t)$	fluorescence intensity detected in the acceptor channel upon excitation at the donor wavelength
$I^{DexDem}(t)$	fluorescence intensity detected in the donor channel upon excitation at the donor wavelength
I_A	steady-state fluorescence intensity of passive acceptors
I_D	steady-state fluorescence intensity of passive donors
I_A^{FRET}	steady-state fluorescence intensity of active acceptors
I_D^{FRET}	steady-state fluorescence intensity of active donors
I_{AM}	steady-state fluorescence intensity of acceptors
I_{DM}	steady-state fluorescence intensity of donors
K_d	dissociation constant between the binding partners
k_{FRET}	FRET transfer rate between donor and acceptor
k_{nrA}	non-radiative decay rate of acceptor
k_{nrD}	non-radiative decay rate of donor
k_{rA}	radiative decay rate of acceptor
k_{rD}	radiative decay rate of donor
$N_A(t)$	excited-state population of unbound acceptors
$N_D(t)$	excited-state population of unbound donors
$N_A^*(t)$	excited-state population of bound acceptors
$N_D^*(t)$	excited-state population of bound donors
$N_A^{tot}(t)$	excited-state population of the total acceptors
$N_D^{tot}(t)$	excited-state population of the total donors
n_A	total number of unbound acceptors
n_D	total number of unbound donors
n_A^*	total number of bound acceptors
n_D^*	total number of bound donors
α_A^{FRET}	intensity contribution from active acceptors
α_D^{FRET}	intensity contribution from active donors
$\mathcal{B}_A^{Dex}$	Einstein stimulated absorption coefficients for acceptor upon donor excitation
$\mathcal{B}_D^{Dex}$	Einstein stimulated absorption coefficients for donor upon donor excitation
β	ratio between $\mathcal{B}_D^{Dex}$ and $\mathcal{B}_A^{Dex}$
$\vec{\tau}_A$ and τ_A	phasor and lifetime of passive acceptor
$\vec{\tau}_A^{FRET}$	phasor of active acceptor
$\vec{\tau}_D$ and τ_D	phasor and lifetime of passive donor
$\vec{\tau}_D^{FRET}$ and τ_D^{FRET}	phasor and lifetime of active donor
$\vec{\tau}_{AM}$	phasor representing fluorescence from the acceptor only (containing signals from active and passive acceptors)
$\vec{\tau}_{DA}$	phasor of fluorescence detected by the FRET channel (containing signals from active and passive acceptors, and signals from active and passive donors)

$\vec{\tau}_{DM}$	phasor for the signal detected by the donor channel (containing signals from active and passive donors)
$\vec{\tau}_{DM}^{FRET}$	phasor for the donor channel signal at vast acceptor excess (taking into account the FRET efficiency distribution and sample impurity)
ψ_A^{Aem}	fraction of acceptor photons collected by the detector in the acceptor emission channel
ψ_D^{Aem}	fraction of donor photons collected by the detector in the acceptor emission channel
ψ_D^{Dem}	fraction of donor photons collected by the detector in the donor emission channel

SUPPORTING REFERENCES:

1. Digman, M. A., V. R. Caiolfa, M. Zamai, and E. Gratton, 2008. The phasor approach to fluorescence lifetime imaging analysis. *Biophys. J.* 94:L14–L16.
2. Colyer, R. A., O. H. Siegmund, A. S. Tremsin, J. V. Vallerga, S. Weiss, and X. Michalet, 2012. Phasor imaging with a widefield photon-counting detector. *J. Biomed. Opt.* 17:0160081–01600812.
3. Visser, N. V., J. W. Borst, M. A. Hink, A. van Hoek, and A. J. Visser, 2005. Direct observation of resonance tryptophan-to-chromophore energy transfer in visible fluorescent proteins. *Biophys. Chem.* 116:207–212.
4. Lakowicz, J. R., 2006. Principles of fluorescence spectroscopy. Springer US.
5. Elder, A., A. Domin, G. K. Schierle, C. Lindon, J. Pines, A. Esposito, and C. Kaminski, 2009. A quantitative protocol for dynamic measurements of protein interactions by Förster resonance energy transfer-sensitized fluorescence emission. *J. R. Soc. Interface.* 6:S59–S81.
6. Kaminski, C. F., E. J. Rees, and G. S. K. Schierle, 2014. A Quantitative Protocol for Intensity-Based Live Cell FRET Imaging. *In* Fluorescence Spectroscopy and Microscopy, Humana Press, 445–454.
7. Stringari, C., A. Cinquin, O. Cinquin, M. A. Digman, P. J. Donovan, and E. Gratton, 2011. Phasor approach to fluorescence lifetime microscopy distinguishes different metabolic states of germ cells in a live tissue. *Proceedings of the National Academy of Sciences* 108:13582–13587.
8. Schlachter, S., A. Elder, A. Esposito, G. Kaminski, J. Frank, L. Van Geest, and C. Kaminski, 2009. mhFLIM: resolution of heterogeneous fluorescence decays in widefield lifetime microscopy. *Opt. Express* 17:1557–1570.
9. Fereidouni, F., G. A. Blab, and H. C. Gerritsen, 2014. Phasor based analysis of FRET images recorded using spectrally resolved lifetime imaging. *Methods Appl. Fluoresc.* 2:035001.
10. Martynov, V. I., B. I. Maksimov, N. Y. Martynova, A. A. Pakhomov, N. G. Gurskaya, and S. A. Lukyanov, 2003. A purple-blue chromoprotein from Goniopora tenuidens belongs to the DsRed subfamily of GFP-like proteins. *J. Biol. Chem.* 278:46288–46292.
11. Zagranichny, V. E., N. V. Rudenko, A. Y. Gorokhovatsky, M. V. Zakharov, Z. O. Shenkarev, T. A. Balashova, and A. S. Arseniev, 2004. zFP538, a yellow fluorescent protein from coral, belongs to the DsRed subfamily of GFP-like proteins but possesses the unexpected site of fragmentation. *Biochemistry.* 43:4764–4772.
12. Medepalli, K., B. W. Alphenaar, R. S. Keynton, and P. Sethu, 2013. A new technique for reversible permeabilization of live cells for intracellular delivery of quantum dots. *Nanotechnology* 24:205101.
13. Xia, Y., and G. M. Whitesides, 1998. Soft lithography. *Annu. Rev. Mater. Sci.* 28:153–184.
14. Devenish, S. R., M. Kaltenbach, M. Fischlechner, and F. Hollfelder, 2013. Droplets as Reaction Compartments for Protein Nanotechnology. *In* Protein Nanotechnology, Springer, 269–286.
15. Gielen, F., T. Buryska, L. V. Vliet, M. Butz, J. Damborsky, Z. Prokop, and F. Hollfelder, 2015. Interfacing Microwells with Nanoliter Compartments: A Sampler Generating High-Resolution Concentration Gradients for Quantitative Biochemical Analyses in Droplets. *Analytical chemistry* 87:624–632.
16. Chan, F. T., G. S. K. Schierle, J. R. Kumita, C. W. Bertoncini, C. M. Dobson, and C. F. Kaminski, 2013. Protein amyloids develop an intrinsic fluorescence signature during aggregation. *Analyst.* 138:2156–2162.
17. Wahl, M., 2014. Time-Correlated Single Photon Counting. Technical report, PicoQuant GmbH, Berlin, Germany.
18. Leray, A., C. Spriet, D. Trinel, Y. Usson, and L. Héliot, 2012. Generalization of the polar representation in time domain fluorescence lifetime imaging microscopy for biological applications: practical implementation. *J. Microsc.* 248:66–76.
19. Szmacinski, H., V. Toshchakov, and J. R. Lakowicz, 2014. Application of phasor plot and autofluorescence correction for study of heterogeneous cell population. *J. Biomed. Opt.* 19:046017–046017.
20. Hinde, E., M. A. Digman, C. Welch, K. M. Hahn, and E. Gratton, 2012. Biosensor Förster resonance energy transfer detection by the phasor approach to fluorescence lifetime imaging microscopy. *Microsc. Res. Tech.* 75:271–281.
21. Laine, R., D. W. Stuckey, H. Manning, S. C. Warren, G. Kennedy, D. Carling, C. Dunsby, A. Sardini, and P. M. French, 2012. Fluorescence Lifetime Readouts of Troponin-C-Based Calcium FRET Sensors: A Quantitative Comparison of CFP and mTFP1 as Donor Fluorophores. *PLoS One.* 7:e49200.
